# Internally diketopyrrolopyrrole-bridged bis-anthracene macrocycle: a multifunctional fluorescent platform†

**DOI:** 10.1039/d4sc06067a

**Published:** 2024-12-03

**Authors:** Huan Zhou, Yuxuan Zhang, Zhiye Zheng, Junhua Wan, Hui Zhang, Kunhua Lin, Jonathan L. Sessler, Hongyu Wang

**Affiliations:** a Department of Chemistry, College of Science, Center for Supramolecular Chemistry & Catalysis, Shanghai University 99 Shangda Road Shanghai 200444 P. R. China wanghy@shu.edu.cn; b College of Material Chemistry and Chemical Engineering, Key Laboratory of Organosilicon Chemistry and Material Technology of Ministry of Education, Hangzhou Normal University Hangzhou P. R. China wan_junhua@hznu.edu.cn; c Laboratory for Microstructures, Instrumental Analysis and Research Center of Shanghai University Shanghai 200444 P. R. China; d Department of Chemistry, The University of Texas at Austin 105 E. 24th Street A5300 Austin TX 78712 USA sessler@cm.utexas.edu

## Abstract

A covalently bridged macrocycle (5) comprising two anthracene strands connected at the lactam positions of a diketopyrrolopyrrole (DPP) chromophore has been constructed. The crystal structure reveals that the central DPP chromophore is wrapped with the externally twisted bis-anthracene macrocycle. The internally bridged macrocycle architecture endows 5 with multifunctional properties. Due to shielding by the double anthracene straps, 5a and a polymer derived from it, DPP-Cycle, display strong fluorescence emission features in both organic media and the solid state. Moreover, the emission colors of these macrocyclic materials can be effectively tuned through external stimuli such as mechanical and thermal treatments, as well as solvent fuming. Compound 5a is stable in the presence of most metal cations but degrades rapidly when it comes in contact with Cu^2+^ in acetonitrile. This decomposition, which is thought to involve a reaction at the central DPP *via* a radical-mediated mechanism, was found to be accelerated in 5a compared to the non-cyclic analogue 2a. This leads us to suggest that internally bridged macrocycles, such as those described here, may have a role to play as fluorescent Cu^2+^ sensors. Finally, the high fluorescence of 5a in the solid state enables its use in the area of latent fingerprint (LFP) imaging.

## Abstract 2

A covalently bridged macrocycle exhibits multifunctional properties, including dual-state emission, intramolecular energy transfer, Cu^2+^ sensing, and mechanochromism, while permitting latent finger print imaging.

## Graphical abstract

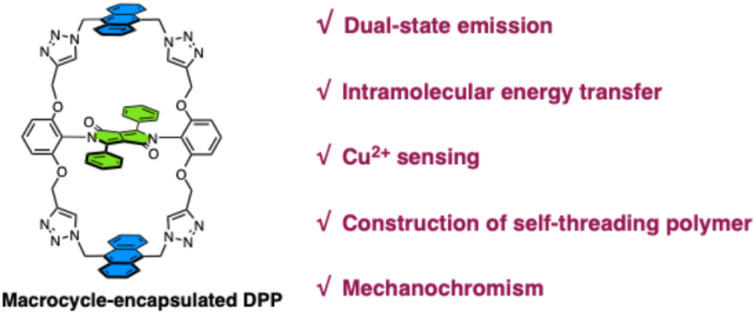

## Introduction

Macrocyclic molecules have played a critical role in the development of supramolecular chemistry.^1^ Increasing efforts have been made to create macrocyclic systems of high complexity or endowed with special functionality. One area where this has been proven fruitful involves the complexation of intrinsically emissive guests.^2–4^ In the best-case scenarios, macrocyclic encapsulation isolates the π-conjugated backbones and can suppress aggregation-caused quenching (ACQ). Typically, this translates into a high level of fluorescence emission in the solid state.^5,6^ Enhanced room-temperature phosphorescence effects (*e.g.*, increased lifetimes and greater phosphorescence quantum efficiencies) can also result from macrocycle encapsulation.^7–9^ Although the formation of complexes containing π-conjugated guests surrounded by insulating macrocyclic hosts is relatively straightforward, ideally requiring only a simple mixing of the host and guest, the resulting supramolecular constructs exist in equilibrium with their constituent components. This can be a limitation in certain applications.

One approach to address the issue of potential complex instability involves covalent linkage of a π-conjugated core to form rotaxane-like architectures, that is, internal π-conjugated molecules are covalently bridged to the external macrocycle.^10–13^ In 2010, Takeuchi *et al.* pioneered the synthesis of a self-threading polythiophene, whose conjugated backbone (*i.e.*, polythiophenes) was sheathed within its own encircling side chains.^14^ The covalently linked alkyl macrocycles could enhance the effective conjugation length of the interior polythiophene backbone. Subsequently, this same research group reported a red-emitting self-threading polymer that achieved a solid-state fluorescence quantum yield (*Φ*_F_) of 13%. Bronstein *et al.* reported a series of fluorescent chromophore-based polymers wrapped with double alkyl straps.^15–20^ They found that the external covalent macrocycles could effectively suppress inter- and intramolecular aggregation, decrease energetic disorder, and increase the backbone collinearity. The most notable of these encapsulated polymers exhibited solid-state quantum yields as high as 41%.^16^ In 2021, Würthner and collaborators described perylene bisimide (PBI) moieties encapsulated within cyclo[*n*]oligothiophenes.^21,22^ It was suggested that the resulting encapsulated donor–acceptor (D–A) dyads benefited from ultrafast Förster resonance energy transfer and photoinduced electron transfer processes. In 2023, Babu *et al.* reported half- and full-oligothiophene-ring-strapped PBIs substituted at the bay positions, and demonstrated ultrafast charge separation and stabilization within these D–A macrocycle dyads.^23,24^ More recently, Wei, *et al.* reported a series of water-soluble double cavity cyclophanes, consisting of central PBI or naphthalene diimide (NDI) cores sheathed by bilateral cationic bipyridinium straps.^25,26^ The resulting constructs displayed excellent near-infrared photothermal effects. In spite of this progress, the number of examples where a π-conjugated molecule benefits from covalent encapsulation with functional macrocycles (as opposed to simply alkyl-based macrocycles) remains limited. Here, we report a covalently macrocycle-wrapped architecture (5) wherein two anthracene units are connected at the lactam positions of a DPP chromophore. A corresponding polymeric version, DPP-Cycle, has also been prepared. As detailed below, these systems display excellent photoluminescence features and can act as rudimentary Cu^2+^ sensors. Moreover, the emission colours of 5 and DPP-Cycle can be tuned effectively through external stimuli, such as grinding, heating, and solvent fuming. Finally, in preliminary work, DPP-Cycle was found to be effective in the area of latent fingerprint (LFP) imaging. Secondary level LFP information could be obtained when a copper plate was used as the substrate and the resulting image was subjected to digital magnification. To the best of our knowledge, LFP imaging is an application that has yet to considered in the context of covalently wrapped chromophore systems.

DPP is one of the most widely used building blocks to construct donor–acceptor conjugated polymers.^27–29^ Like most planar conjugated molecules, DPP suffers from ACQ.^30^ As a result, it exhibits a high *Φ*_F_ in dilute solution, but a very low *Φ*_F_ in the solid state. Inspired by recently reported covalently bridged cyclic structures and our previous work on DPP,^31–34^ we hypothesized that the ACQ effects could be suppressed by creating a doubly anthracene-strapped DPP 5 wherein the DPP serves as the molecular axis of a rotaxane-type structure. This study was undertaken in an effort to test this hypothesis.

## Results and discussion

### Synthesis

**Scheme 1:**
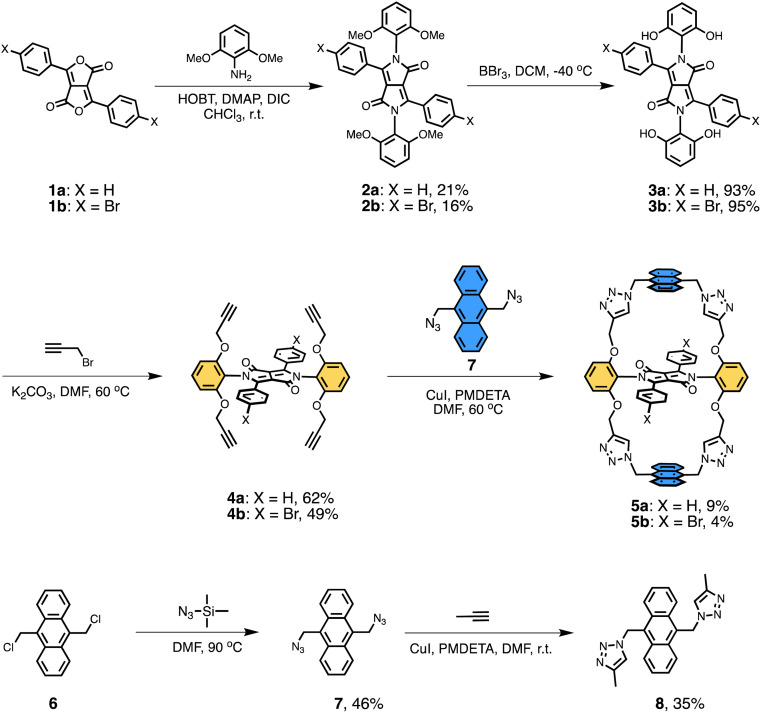
Synthesis of doubly anthracene-strapped diketopyrrolopyrrole 5 and model compound 8.

The synthetic route to the doubly anthracene-strapped DPP macrocycles of this study (5a and 5b), as well as model compounds 2a and 8, are outlined in Scheme 1. Full synthetic procedures for 5a, 5b, and preparative intermediates are provided in the ESI.† Briefly, compounds 1a and 1b, used as starting materials, were prepared according to previously reported procedures.^35^ They were then reacted with 2,6-dimethoxyaniline in the presence of 1-hydroxybenzotriazole hydrate (HOBT), 4-(dimethylamino)pyridine (DMAP), and *N*,*N*′-diisopropylcarbodiimide (DIC) at room temperature for seven days to afford the corresponding *N*-arylated products 2a and 2b in yields of 21% and 16%, respectively. Subsequent treatment of 2a and 2b with BBr_3_ in dichloromethane provided the tetrahydro-DPP derivatives 3a and 3b, which were subjected to a base-mediated nucleophilic reaction with propargyl bromide to afford the tetraalkynyl-DPPs 4a and 4b in yields of 62% and 49%, respectively. Finally, a copper(i)-catalysed azido-alkyne cycloaddition (CuAAC) reaction of 4a and 4b with 9,10-bis(azidomethyl)anthracene 7 produced the target doubly anthracene-strapped DPP macrocycles 5a and 5b in yields of 9% and 4%, respectively. The triazole–anthracene–triazole model compound 8 was also prepared for comparison. The structures of all new compounds were confirmed by ^1^H NMR and ^13^C NMR spectroscopy and ESI-TOF-MS (Fig. S1–S22†), as well as by single crystal X-ray diffraction analysis for 2a and 5a (see below).

### Crystal structures

**Fig. 1:**
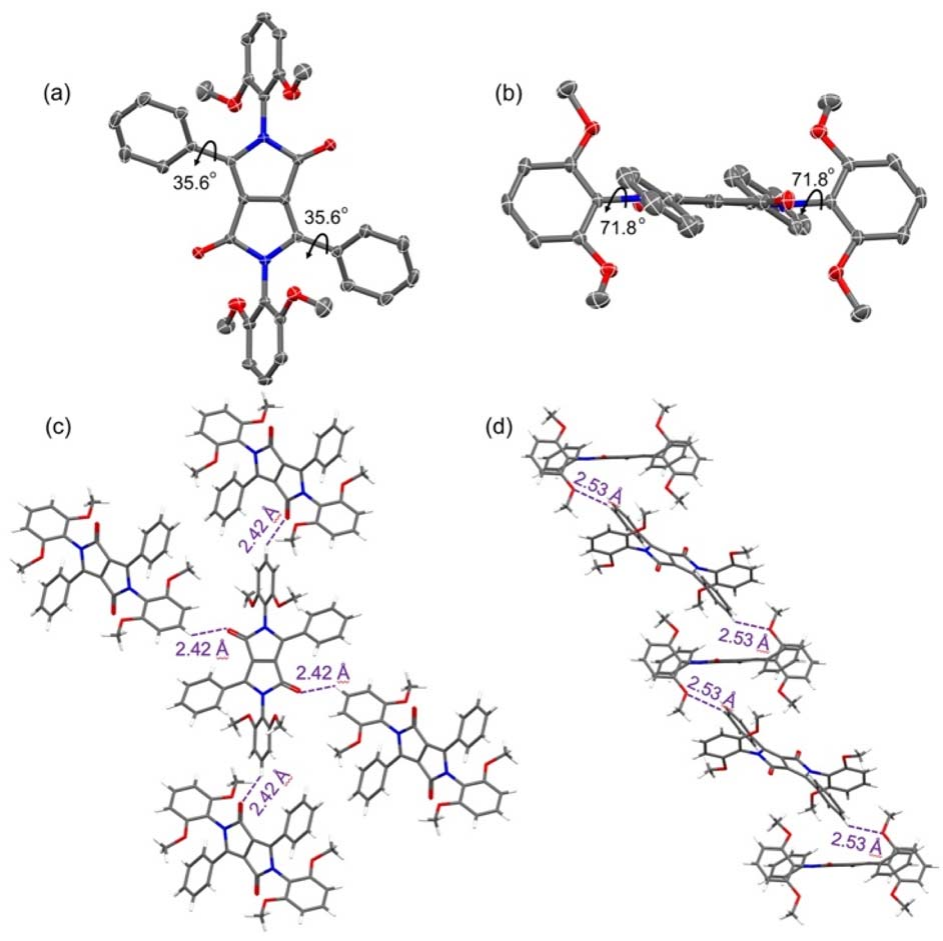
ORTEP drawing of compound 2a with the thermal ellipsoids set at the 50% probability level. Atom color codes: C, grey; N, blue; O, red: (a) front view, (b) side view, (c) and (d) intermolecular interactions between adjacent molecules. The purple dashed line represents inferred hydrogen bond interactions between adjacent molecules. Solvent molecules and hydrogen atoms with partial occupancy are omitted for clarity.

Diffraction grade crystals of the DPP reference compound 2a were obtained *via* the slow diffusion of methanol vapor into a chloroform solution of 2a (Fig. 1, S23 and Table S1†). The resulting structure confirmed the presence of four phenyl groups. The two flanking phenyl groups are rotated by 35.6° with respect to the central DPP core. The other two phenyl groups, linked at the lactams, are nearly perpendicular to the DPP core with identical dihedral angles of 71.8°. The net result is a twisted conformation that is expected to provide partially shielding of the DPP core. Specifically, the built-in steric hindrance provided by the phenyl substituents was expected to prevent intermolecular π–π stacking. As expected, no close π–π stacking involving the DPP cores is observed in the crystal packing diagram. On the other hand, two types of hydrogen bonds between the neighbouring molecules were observed (Fig. 1c and d). One set of interactions (with H⋯O distances of 2.42 Å) is between the phenyl hydrogen atoms and carbonyl oxygen atoms. The other interactions are between the hydrogen atoms of the phenyl groups and the methoxy oxygen atoms and are characterized by H⋯O distances of 2.53 Å. The net result is a staggered arrangement as previously reported for PBI derivatives bearing bulky substituents at the imide positions.^36,37^

**Fig. 2:**
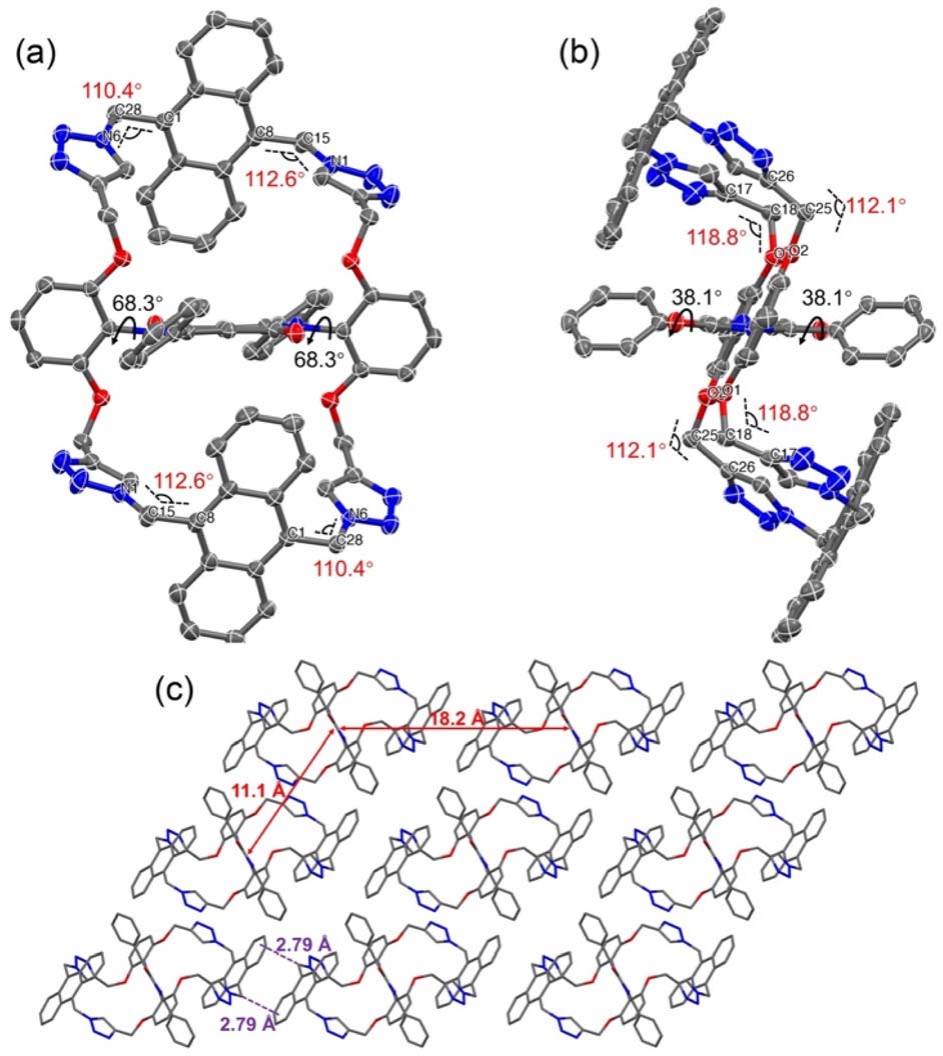
ORTEP drawing of compound 5a with the thermal ellipsoids set at the 50% probability level. Atom color codes: C, grey; N, blue; O, red. (a) Front view, (b) side view, and (c) molecular packing diagram. The red solid lines represent the distance between adjacent DPP cores, and the purple dashed line represents the interplanar distance between the adjacent anthracenes. Hydrogen atoms and solvent molecules are omitted for clarity.

An X-ray crystallographic analysis of a single crystal of 5a, obtained *via* the slow diffusion of methanol vapor into an acetonitrile solution of 5a, confirmed the expected doubly anthracene-strapped macrocyclic structure (Fig. 2, S24 and Table S1†). A twisted Z-shaped conformation is observed, with both anthracene-strapped macrocycles adopting a geometry that appears to minimize distortion with respect to the central DPP plane. This effect is manifested at the carbon atoms adjacent to the triazole rings with torsional angles of 112.1° and 118.8° associated with atoms C17–C18–O1 and C26–C25–O2, respectively. The central DPP core is almost planar, and the dihedral angles between the DPP core and its adjacent phenyl units are 38.1°, a value that is almost identical to that of the non-cyclic reference system 2a.^38^ The packing diagram reveals that 5a exists in the form of slipped stacks characterized by an interplanar distance of about 3.47 Å and inferred intermolecular C–H⋯π interactions (2.79 Å) (Fig. 2c). Perhaps reflecting steric shielding by the macrocycles, the minimum distances between the centroids of the DPP cores are approximately 11.1 Å and 18.2 Å. A high degree of spatial separation in thus enforced in the solid state. This stands in contrast to what has been seen in alkyl-substituted DPPs.

### Photophysical properties

**Fig. 3:**
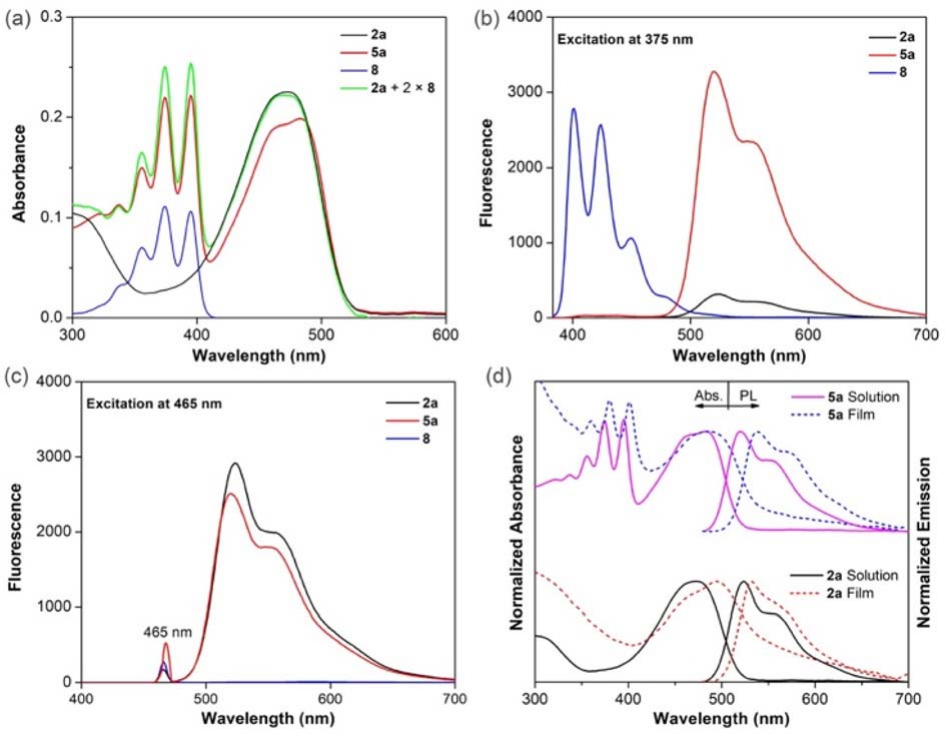
(a) UV-vis absorption spectra of 2a, 5a and 8 in chloroform (10 μM). Fluorescence spectra of 2a, 5a and 8 in chloroform (1 μM) recorded upon excitation of the anthracene chromophore at 375 nm (b) and excitation of the DPP chromophore at 465 nm (c). (d) Normalized UV-vis absorption spectra and emission spectra of 2a and 5a in chloroform solution (solid line) and as thin films (dashed line).

Spectroscopic studies of macrocycle 5a and model compounds 2a and 8, as dilute solutions (10^−5^ M), were carried out in chloroform (Fig. 3 and Table 1). Macrocycle 5a displays characteristic absorptions of both the anthracene and DPP moieties, as highlighted by comparisons with model compounds 2a and 8 (Fig. 3a). In fact, 5a, containing two anthracene subunits and one DPP moiety, gives rise to a spectrum that is close to a linear superposition of the high energy anthracene absorption observed below 400 nm with three well-resolved vibronic progressions and a low energy broad absorption from the DPP moiety. Nevertheless, compared to what would be expected for this linear superposition, a slight decrease in the absorption intensity of 5a can be discerned; presumably this reflects the conformational restriction imposed by the macrocycle. On the basis of these studies, we conclude that there is little, if any, ground state electronic interaction between the anthracene subunits and the DPP core.

**Table 1:** Absorption and fluorescence properties of model compound 2a, macrocycle 5a, and the corresponding polymers

Sample	*λ*_abs_ (nm)	*λ*_em,max_ (nm)	*Φ*_F_ a (%)	*τ* (ns)
2a in CHCl_3_	471	523	88.8 ± 1.8	5.51^b^
				5.71^c^
2a film	494	532	4.6 ± 1.2	*τ*_1_ = 0.55 (57.4%)
				*τ*_2_ = 1.96 (42.6%)
5a in CHCl_3_	356, 374, 395, 483	520	98.4 ± 1.6	6.46^b^
				6.65^c^
5a film	360, 380, 401, 486	537	10.1 ± 0.2	*τ*_1_ = 0.73 (79.0%)
				*τ*_2_ = 4.27 (21.0%)
DPP-C8C12 in TCB	407, 519	597	44.7 ± 0.52	2.56^b^
DPP-C8C12 film	407, 525	663	7.7 ± 0.6	*τ*_1_ = 1.28 (22.8%)
				*τ*_2_ = 5.01 (77.2%)
DPP-Cycle in TCB	378, 399, 518	596	43.8 ± 0.24	2.23^b^
DPP-Cycle film	402, 475, 518	622	15.7 ± 1.4	*τ*_1_ = 0.86 (66.2%)
				*τ*_2_ = 2.30 (33.8%)

a Measured using an integrating sphere. The solution phase studies were carried out in chloroform or 1,2,4-trichlorobenzene (TCB) as dilute solutions, whereas thin films were spin-coated from chlorobenzene solution.

b Excitation at 375 nm.

c Excitation at 465 nm.

Due to its wrapped structure, 5a was expected to benefit from intramolecular energy/electron transfer between anthracene (donor) and DPP (acceptor) moieties. To test this proposition, the emission spectra of macrocycle 5a were recorded upon selective excitation of the anthracene and DPP absorption spectral regions. Upon selective excitation of the anthracene moieties at 375 nm, little emission is seen that can be ascribed to anthracene fluorescence. In contrast, under these conditions, the fluorescence intensity of the DPP moiety is increased by about 10-fold compared to model compound 2a excited under otherwise identical conditions (Fig. 3b). This is taken as evidence of efficient intramolecular energy transfer from the bilateral anthracene donors to the central DPP acceptor. It is important to note that the emission spectrum of anthracene overlaps with the absorption spectrum of DPP (Fig. S25†). The relative ratio of the peaks corresponding to the anthracene donor, normalized to the peaks corresponding to the DPP acceptor, was used to estimate the energy transfer efficiency. According to this method, the energy transfer from anthracene moieties to the DPP moiety is estimated to be approximate 90% (Fig. S26†). When subjected to photoexcitation at 465 nm (DPP spectral region), both 5a and 2a show almost the same characteristic vibronic fine structures with the emission maximum at 519 nm and a shoulder at about 556 nm (Fig. 3c).^32,39^ The emission intensity of 5a is, however, slightly smaller than that of 2a, a finding that may reflect the smaller absorption at 465 nm by the DPP moiety in 5a as compared to 2a.

Next, the optical properties of 2a and 5a were studied in thin films. To a first approximation, the solution phase features were retained in the thin films, although with redshifts of approximately 9 and 17 nm, respectively (Fig. 3d). Additionally, the absorption spectrum of 5a shows a steeper onset in the thin film compared to 2a, a finding interpreted as indicating that 5a possesses a lower level of conformational disorder in the ground state.

As expected, 2a and 5a displayed high fluorescence quantum yields in dilute chloroform solution, with the *Φ*_F_ for 5a being close to unity. In contrast, in the solid state, 2a exhibits the effect of presumed ACQ fluorescence quenching as indicated by a *Φ*_F_ value of only 4.6%. A larger quantum yield (*Φ*_F_ = 10.1%) was seen in the case of 5a. This contrast in values is consistent with the design expectation that the introduction of an external macrocycle can prevent aggregation between fluorescent chromophores and partially suppress ACQ effects.^15–19^

### Metal ion sensing

**Fig. 4:**
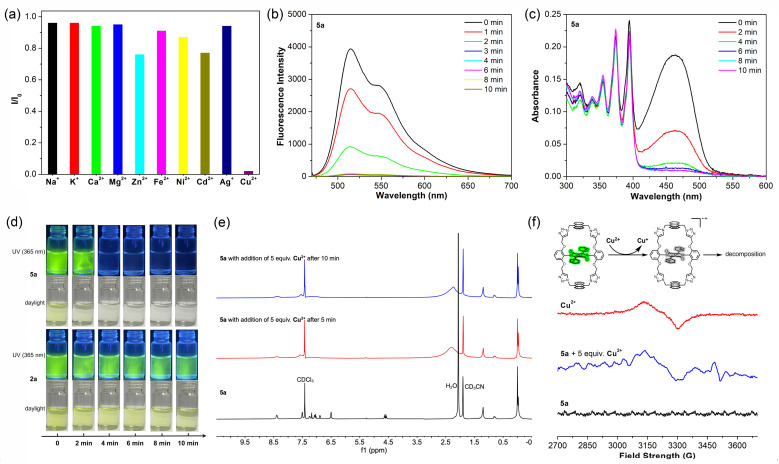
(a) Relative fluorescence intensities of 5a (10^−5^ M, in CH_3_CN) as seen in the presence of various metal ions including trifluorosulfonate salts of Na^+^, K^+^, Ca^2+^, Mg^2+^, Zn^2+^, Fe^2+^, Ni^2+^, Ag^+^, and Cu^2+^ and perchlorate salt of Cd^2+^. Except for Cu^2+^, which was added at 5.0 equiv., other metal ions were added at the 100 equiv. level. Time dependent changes in the fluorescence intensity (b) and UV-vis absorbance features (c) of 5a (10^−5^ M) recorded after the addition of 5 equiv. of Cu^2+^ in acetonitrile. (d) Comparison of the color changes induced when acetonitrile solutions of 2a and 5a were treated with 5 equiv. of Cu^2+^. (e) Changes in the ^1^H NMR spectrum of 5a seen before and after the addition of Cu^2+^ in deuterated acetonitrile (containing 25% CDCl_3_). (f) EPR spectra of Cu^2+^ (10 mM) and 5a (5 × 10^−4^ M) with or without 5 equiv. of Cu^2+^ in acetonitrile at room temperature. Inset: proposed decomposition mechanism *via* a radical cation process. See the main text for discussion.

Given the presence of two geometrically defined cavities and the abundance of likely metal coordination sites, macrocycle 5a in acetonitrile (10^−5^ M) was treated with a variety of common metal ions and the resulting fluorescence response, if any, was recorded. As shown in Fig. 4a, as well as S27 and S28,† addition of 100 equivalents of Na^+^, K^+^, Ca^2+^, Mg^2+^, Zn^2+^, Fe^2+^, Ni^2+^, Cd^2+^ and Ag^+^ (trifluorosulfonate salts, except for Cd^2+^, which was used in the form of its perchlorate salt) had a negligible effect on either the UV-vis absorption or fluorescence spectra of 5a. In contrast, only 5 equivalents of Cu^2+^ was found to quench almost completely the fluorescence of 5a (Fig. 4b). No bathochromic shift of the emission bands or new emission bands were observed. Furthermore, the UV-vis absorption spectra of 5a, recorded upon the addition of Cu^2+^ revealed that stepwise addition of Cu^2+^ leads to a gradual decrease in the intensity of the DPP moiety absorption (420–520 nm) before it finally disappears. In contrast, no significant change in the anthracene absorption features (330–410 nm) was observed (Fig. 4c). These results are taken as evidence, that the Cu^2+^ interacts directly with the DPP core, but not with the anthracene subunits. In a comparison study carried out under otherwise identical conditions, a solution of 2a was titrated with Cu^2+^. In this case, only partial quenching of the fluorescence emission of 2a was seen after 10 min (Fig. S29 and S30†). These changes could also be observed visually (Fig. 4d). After adding Cu^2+^, the initial light-yellow colour of the solution of 5a became colourless within 4 min. The initial strong green luminescence of solutions of 5a was also found to fade gradually to the point of being unobservable under a 365 nm UV lamp. The colour of the corresponding solution of 2a was found to fade much more slowly compared to 5a, with fluorescence still being readily observable after 10 min.

The effects of Cu^2+^ on the fluorescence features of 5a led us to explore its possible use as a divalent copper sensor. Formation of Cu^2+^–ligand complexes is arguably the most common mechanism for Cu^2+^ sensing.^40–42^ Although often benefiting from high specificity, Cu^2+^–ligand complexes typically used for sensing are often labile and can be dissociated by treating with molecules displaying stronger affinities for Cu^2+^, such as ethylene diamine tetraacetic acid (EDTA).^43^ When EDTA was added to premixed solutions of 2a and Cu^2+^ or 5a and Cu^2+^, the Cu^2+^-induced alterations in the absorption and fluorescence features of the DPP moiety were not recovered (Fig. S31–S34†). On this basis, we conclude that the spectral changes discussed above do not reflect the formation of 2a–Cu^2+^ and 5a–Cu^2+^ complexes.

To gain insights into the events associated with treating 2a and 5a with Cu^2+^, the ^1^H NMR spectra were recorded before and after the addition of Cu^2+^ (Fig. 4e and S35†). It was found that the characteristic aromatic peaks of 5a decreased in intensity and disappeared roughly 5 min after adding 5 equivalents of Cu^2+^. In the case of 2a, exposure to 5 equivalents of Cu^2+^ caused a decrease in the aromatic peaks, which then became difficult to discern clearly after 10 min. The MS spectrum of 5a is characterized by the presence of two peaks at *m*/*z* = 1233.23 and 1256.24 corresponding to [5a + H]^+^ and [5a + Na]^+^, respectively (Fig. S13†). Neither peak was observed following the addition of Cu^2+^ (Fig. S36†). This leads us to suggest that interaction with Cu^2+^ in acetonitrile results in decomposition of the DPP core in both 2a and 5a, with the macrocyclic nature of the latter system apparently serving to accelerate the decomposition process.

Aromatic amines have been reported to easily form radicals in acetonitrile in the presence of Cu^2+^.^44,45^ Recently, Liang *et al.* reported that the decomposition of an *N*-alkyl disubstituted DPP could be triggered by Cu^2+^ in acetonitrile through a suggested radical mechanism.^46^ We thus propose that the *N*-aryl disubstituted DPP in 2a and 5a could also promote the formation of radicals in acetonitrile in the presence of Cu^2+^. Formation of a DPP radical would then be a key step leading ultimately to the decomposition of the DPP core. This decomposition would then be evident through readily discernible changes in the optical properties.

Electron paramagnetic resonance (EPR) spectroscopy is a time-honored technique used to detect free radicals in chemical and biological systems. It was thus used to monitor putative radical processes associated with presumed DPP decomposition. In these studies, the focus was on 5a over 2a due to solubility considerations and the more dramatic nature of the Cu^2+^-induced effect in acetonitrile. As shown in Fig. 4f, a horizontal line can be observed in the EPR spectrum of pure 5a solution (5 × 10^−4^ M, in CH_3_CN), indicating the absence of radical species. As expected, a broad EPR peak is seen for pure Cu^2+^ (10 mM, in CH_3_CN) reflecting its paramagnetic nature. Upon the addition of 5 equivalents of Cu^2+^ to a solution of 5a, a new, weak EPR signal appeared at 3500 G, which was attributed to the formation of the DPP radical concurrent with the reduction of Cu^2+^ to Cu^+^. This new signal was concordant with the DPP radical EPR spectrum reported previously by Liang *et al.*^46^ We thus suggest that the DPP unit of 5a decomposes when it comes in contact with Cu^2+^ in acetonitrile *via* a radical mechanism. We also suggest that the macrocyclic nature of 5a serves to enhance this effect relative to the non-cyclic analogue, 2a.

### Poly-bicyclic polymers

**Scheme 2:**
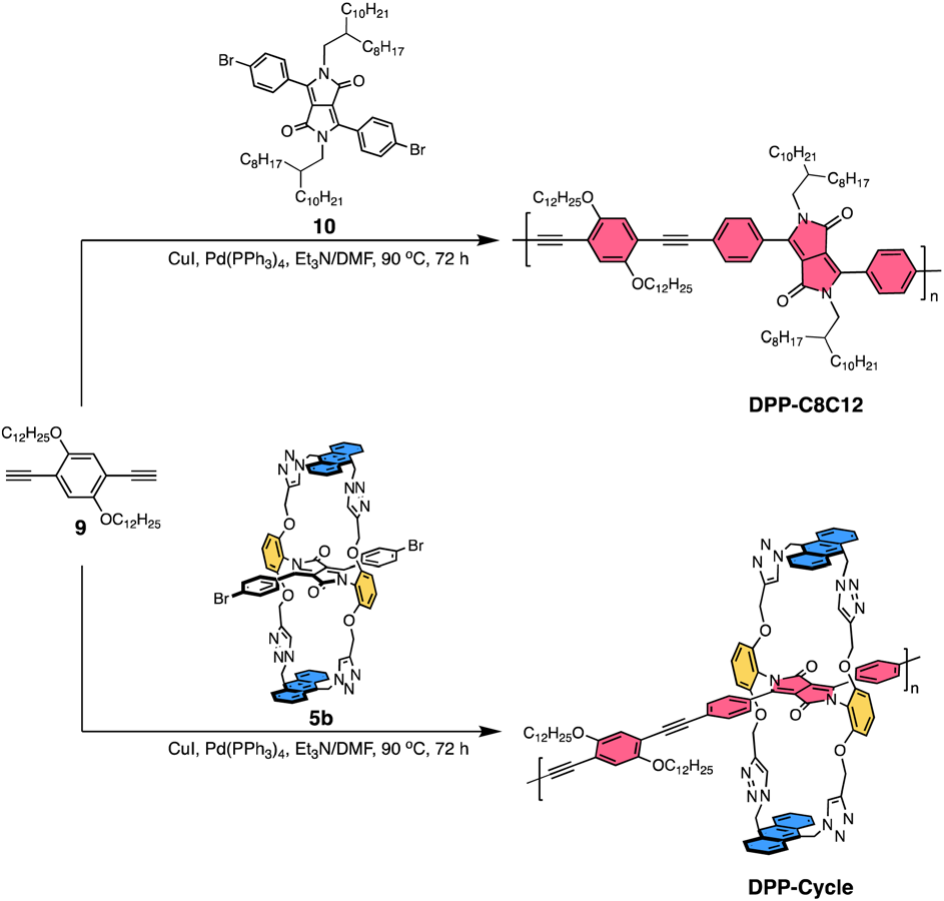
Synthesis of DPP-C8C12 and DPP-Cycle through the Sonogashira cross-coupling reaction.

With monomer 5b in hand, a poly-bicyclic polymer DPP-Cycle was synthesized by means of a palladium-catalyzed Sonogashira–Hagihara cross-coupling copolymerization using 1,4-bis(dodecyloxy)-2,5-diethynylbenzene 9 as the co-monomer. The non-cyclic analogue, DPP-C8C12, a compound bearing branched 2-octyldodecyl substituents, was synthesized for comparison (Scheme 2). Both polymers feature the same backbone, but different sidechains attached at the DPP monomers. The polymers were purified by successive Soxhlet extraction with methanol, acetone, hexanes, and chloroform in sequence. The resulting leachates were collected and the volatiles were removed under reduced pressure to afford the corresponding polymers. These polymers exhibited moderate solubility in solvents such as chlorobenzene (CB) and 1,2,4-trichlorobenzene (TCB). The number average molecular weights (*M*_n_) and weight average molecular weights (*M*_w_) were 25.5 and 37.1 kg mol^−1^ for DPP-Cycle, and 17.7 and 35.7 kg mol^−1^ for DPP-C8C12, respectively, as determined by gel permeation chromatography (GPC) using 1,2,4-trichlorobenzene (TCB) as the eluent at 150 °C (Table S2†). Thermogravimetric analyses (TGA) of both polymers under an N_2_ atmosphere revealed high thermal stability for both systems with decomposition temperatures (*T*_d_, corresponding to 5% weight loss) greater than 330 °C (Fig. S37†).

**Fig. 5:**
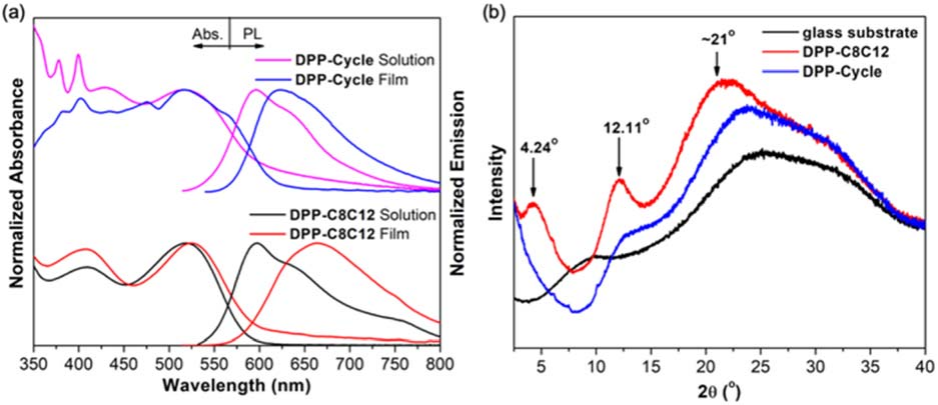
(a) UV-vis absorption and fluorescence spectra of DPP-C8C12 and DPP-Cycle in dilute trichlorobenzene solution and in thin films. (b) XRD patterns of DPP-C8C12 and DPP-Cycle thin films measured on a glass substrate.

The solution and thin film absorption and emission spectra of both polymers are shown in Fig. 5a. The absorption spectral profiles in dilute solution and in the thin films were essentially identical for both DPP-C8C12 and DPP-Cycle; only a modest 6 nm and 1 nm redshift was seen for these two polymers, respectively, upon transitioning from solution to the thin film state. Both DPP-C8C12 and DPP-Cycle exhibited almost identical fluorescence spectra in solution with a *λ*_em,max_ of about 596 nm and a shoulder at 620 nm. Moreover, similar quantum yields of approximately 44% and *τ* values of 2.5 ns were observed for both polymers. We take this as evidence that the observed fluorescence emission originates from the isolated polymer mainchains in solution, rather than from polymer–polymer interactions, which would be expected to vary as a function of structure. A different behavior was observed in the thin films. For instance, the *λ*_em,max_ of DPP-C8C12 red-shifts from 597 to 663 nm upon transitioning from solution into a thin film. A smaller red-shift in the *λ*_em,max_ (by only about 26 nm, *i.e.*, from *λ*_em,max_ = 596 nm to 622 nm) is observed for DPP-Cycle when studied as a film. The thin film *Φ*_F_ values for DPP-C8C12 and DPP-Cycle were 7.7% and 15.7%, respectively. This finding is easily rationalized in terms of macrocyclic shielding (as in DPP-Cycle), which serves to suppress the aggregation of the polymer mainchains more effectively than the branched long alkyl chains present in DPP-C8C12. This conclusion corroborates previous findings involving encapsulated conjugated materials.

X-ray diffraction (XRD) analyses were performed to determine the crystallinity of the polymer thin films on a glass substrate (Fig. 5b). It was found that DPP-Cycle is essentially amorphous as inferred from the absence of prominent reflection peaks. In contrast, DPP-C8C12 as a thin film exhibited several moderate reflection peaks, respectively, consistent with the formation of a well-ordered structure.^47,48^ The peak centred at 4.24°, corresponding to a *d*_100_-spacing value of 20.8 Å, is thought to reflect the intermolecular spacing between a pair of main chains separated by long branched side chains. A broad and fuzzy diffraction peak centred at about 20°, overlapping with the scattering from the glass substrate, is also seen. This feature is thought to reflect the face-to-face packing distance within the polymer main chain. Although not analysed in detail the feature at 12.11° is thought to reflect the presence of higher order structures.

### Mechanoluminescence

**Fig. 6:**
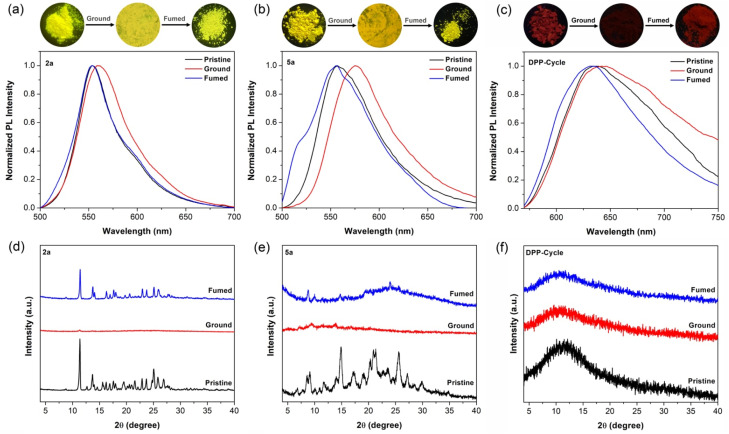
The fluorescence spectra of solid powders of 2a (a), 5a (b) and DPP-Cycle (c) studied in different solid state forms. Top: Corresponding fluorescence images recorded under a 365 nm ultraviolet lamp. The corresponding PXRD patterns of 2a (d), 5a (e) and DPP-Cycle (f) recorded in different solid state forms.

The solid-state optical and electronic properties of organic chromophores are often related directly to the degree of aggregation and the morphologies of the resulting ensembles. Macrocycles that protect internal molecules can reduce the extent of interactions in the solid state, thus leading to enhanced emission-related features. These effects are expected to depend on the specifics of the molecular arrangements in the solid state. This offers the appealing opportunity to tune the molecular aggregation behavior by application of various stimuli, such as mechanical force.^49,50^ The mechanoresponsive luminescence behavior of 2a, 5a and DPP-Cycle was thus studied. The results are summarized in Fig. 6 and Table 2.

**Table 2:** Maximum emission wavelength (*λ*_em_), absolute fluorescence quantum yields (*Φ*_F_) and lifetimes (*τ*) of pristine and ground powder samples

Sample	State	*λ*_em,max_ (nm)	*Φ*_F_ (%)	*τ* (ns)
2a	Pristine	553	23.2 ± 1.3	*τ*_1_ = 2.42 (35.1%)
				*τ*_2_ = 6.03 (64.9%)
	Ground	559	8.0 ± 0.8	*τ*_1_ = 1.98 (61.9%)
				*τ*_2_ = 4.72 (38.1%)
5a	Pristine	557	20.5 ± 1.8	*τ*_1_ = 0.96 (33.8%)
				*τ*_2_ = 2.54 (66.1%)
	Ground	575	4.8 ± 0.7	*τ*_1_ = 0.93 (44.3%)
				*τ*_2_ = 2.98 (55.7%)
DPP-Cycle	Pristine	635	2.0 ± 0.1	*τ*_1_ = 0.68 (62.1%)
				*τ*_2_ = 2.79 (37.9%)
	Ground	642	0.5 ± 0.1	*τ*_1_ = 0.42 (69.1%)
				*τ*_2_ = 3.28 (30.9%)

By concentrating dichloromethane solutions using a rotary evaporator, crystalline powders of 2a and 5a were obtained. The resulting solid species display bright green-yellow fluorescence with emission peaks at 553 and 557 nm, respectively. When pristine 2a crystalline powder was thoroughly ground in an agate mortar, a slight red-shift was observed in its emission (∼6 nm), along with a decrease in the *Φ*_F_ from 23.2% to 8.0%. Similar phenomena were observed for 5a. In this latter case, grinding led to a red-shift in the emission spectral maximum from *λ*_em,max_ = 557 nm (*Φ*_F_ = 20.5%) to *λ*_em,max_ = 575 nm (*Φ*_F_ = 4.8%). These spectral changes correspond well with the variations in emission color observed by the naked eye.

Powder X-ray diffraction (PXRD) analyses of pristine crystalline powders of 2a and 5a revealed sharp and intense peaks, as would be expected for microcrystalline samples. These sharp features disappeared upon grinding, presumably as the result of forming an amorphous state (Fig. 6d and e). On the other hand, solution phase ^1^H NMR spectra of the ground samples of 2a and 5a matched well with those recorded prior to grinding (Fig. S38 and S39†). We thus suggest that the grinding process has little effect on the chemical structure.

The color changes could be reversed to regenerate the pristine, *i.e.*, prior-to-grinding state by solvent fuming and heat-annealing (Fig. S40†). In the case of 2a, samples obtained after grinding completely recovered their fluorescence features, including color, upon exposure to dichloromethane vapor for 20 min. The crystalline state was regenerated as inferred from the fluorescence spectrum and PXRD analyses after fuming (Fig. 6d). Subjecting a ground sample of 5a to fuming with dichloromethane vapor under identical conditions led to significant recovery of the luminescence color. However, a slightly broader emission was observed compared to the pristine powder. Partial recovery of crystallinity was observed after subjecting a ground sample of 5a to dichloromethane fuming, as inferred from PXRD analysis (Fig. 6d).

The non-cyclic polymer, DPP-C8C12, did not show appreciable mechanoresponsive luminescence behavior. However, the bicyclic polymer, DPP-Cycle, exhibited similar stimuli-response luminescence as seen in the case of the monomeric macrocycle 5a. Specifically, grinding results in a slight red-shift in the emission and an obvious decrease in the *Φ*_F_ value. Likewise, solvent fuming and heat-annealing were found to restore largely the photophysical properties to those of the unground form.^51,52^ In this case, PXRD analyses indicated that these changes correlated with an amorphous-to-amorphous conversion (Fig. 6f). While not definitive proof, this is consistent with conversions between several thermodynamically (meta)stable states.

### Latent fingerprint (LFP) imaging

LFPs are imprints left by human fingers on surfaces or substrates. Typically, LFPs contain moisture and grease, as well as absorbed dust. An ability to read LFPs can play a critical role in forensics.^53–55^ In light of its high fluorescence in the solid state, we sought to explore whether 5a could be used for LFP imaging. To test this possibility, a sample of 5a was doped into silica gel (300–400 mesh, 0.1 wt% doping ratio) to enhance its adhesion to LFPs. The resulting powder (5a@silica) was characterized by a strong green fluorescence and demonstrated good stability when dispersed onto silica gel (Fig. S41†). It was thus tested for its ability to facilitate LFP visualization under conditions of powder dusting.

**Fig. 7:**
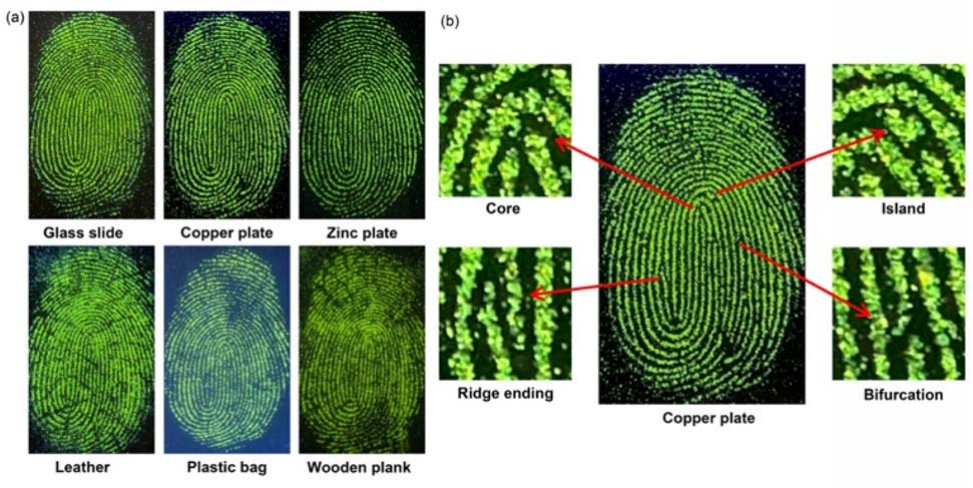
(a) Fluorescent images of LFPs developed using 5a@silica on different substrate surfaces. (b) Secondary level information visible when a copper plate was used as the substrate with the LFP subjected to digital magnification.

Generally, LFP imaging involves a simple three-step process: fingerprint deposition, powder dusting, and fluorescence imaging. To obtain a suitable fingerprint for testing, the fingertips of one coauthor were pressed onto various surfaces, including glass slides, copper plates, zinc plates, leather, transparent plastic bags, and even wooden planks. Subsequently, the 5a@silica powder was carefully sprinkled on the fingerprinted surfaces, and the excess powder was gently blown away using air. The small residual amount of 5a@silica powder adhered to the sebaceous of LFPs was found to emit a strong green fluorescence upon excitation with a UV lamp (365 nm). Excellent contrast between the fluorescent ridges and non-fluorescent furrows was seen under these conditions (Fig. 7a) and was readily apparent to the naked eye. More importantly, the fingerprint details on the surfaces of a glass slide, copper plate and zinc plate can be well resolved at higher magnification. The magnified images exhibit well-defined secondary level fingerprint characteristics, *i.e.*, core, island, ridge ending and bifurcation (Fig. 7b).^54^ It is worth noting that these experiments were carried out using a smartphone and a UV-lamp. Since these devices are portable and easily available, this method may allow for instant outdoor imaging of LFPs on immovable substrates.

## Conclusions

In summary, we have designed and synthesized a multifunctional bridged macrocycle molecule comprising a central DPP core covalently wrapped with two anthracene-containing strands. The external macrocycle provides effective shielding of the central DPP luminescent core, resulting in dual-state emission features in the covalently bridged molecule 5a and its corresponding bicyclic polymer DPP-Cycle. These emission features are partly attributed to steric protection from the environment, as well as efficient photoinduced energy transfer from the bilateral anthracene subunits to the emissive central DPP core upon photoexcitation in the anthracene spectral region. It was found that in acetonitrile solution, Cu^2+^ degrades the DPP chromophore present in 5a through a presumed radical mechanism. The macrocycle 5a was found to degrade faster than the non-cyclic control 2a. Both 5a and its polymeric analogue, DPP-Cycle, displayed stimuli-responsive luminescence in the solid state, which could be influenced by mechanical treatment and solvent fuming. Finally, in preliminary studies, 5a enabled latent fingerprint imaging on various substrate surfaces. On the basis of the results presented here, we suggest that covalently linked macrocycle systems will provide new opportunities in the area of optical and optoelectronic materials.

## Data availability

All data are included in the main text, the ESI,† or (X-ray work only) uploaded to the Cambridge Crystallographic Data Centre.

## Author contributions

The manuscript was written through contributions from all authors. All authors have given approval to the final version of the manuscript. Huan Zhou and Yuxuan Zhang: synthesis, structural characterization, writing – original draft; Zhiye Zheng: crystal growth; Junhua Wan: fluorescence measurements and data analysis; Hui Zhang: fluorescence measurements; Kunhua Lin: supervision and data analysis; Jonathan L. Sessler: supervision and writing – review & editing; Hongyu Wang: resources, project administration, supervision, writing – review & editing.

## Conflicts of interest

There are no conflicts to declare.

## Supplementary Material

SC-OLF-D4SC06067A-s001

SC-OLF-D4SC06067A-s002
