# Supplementary material for: Internally diketopyrrolopyrrole-bridged bis-anthracene macrocycle: a multifunctional fluorescent platform
Source: Chem Sci. 2024 Dec 3;16(2):910–9. doi: 10.1039/d4sc06067a (PMC11626631; doi:10.1039/d4sc06067a)
Supplement: SC-016-D4SC06067A-s001 [file SC-016-D4SC06067A-s001.pdf]

Supplementary Information (SI) for Chemical Science.
This journal is © The Royal Society of Chemistry 2024

Electronic Supplementary Material (ESI) for Chemical Science.

This journal is © The Royal Society of Chemistry 2024

Electronic Supplementary Information

Internally diketopyrrolopyrrole-bridged bis-anthracene macrocycle: A multifunctional fluorescent platform

Huan Zhou,[a] Yuxuan Zhang,[a] Zhiye Zheng,[a] Junhua Wan,*[b] Hui Zhang,[c] Kunhua Lin,*[a] Jonathan L. Sessler *[d] and Hongyu Wang*[a]

[a] Department of Chemistry, College of Science, and Center for Supramolecular Chemistry & Catalysis, Shanghai University, 99 Shangda Road, Shanghai, 200444, P. R. China. E-mail: wanghy@shu.edu.cn

[b] College of Material Chemistry and Chemical Engineering, Key Laboratory of Organosilicon Chemistry and Material Technology of Ministry of Education, Hangzhou Normal University, Hangzhou, P. R. China. E-mail: wan_junhua@hznu.edu.cn

[c] Laboratory for Microstructures, Instrumental Analysis and Research Center of Shanghai University, Shanghai 200444, P. R. China

[d] Department of Chemistry, The University of Texas at Austin 105 E. 24th Street A5300, Austin, TX 78712, USA. E-mail: sessler@cm.utexas.edu

Table of Contents

1. General Experimental..........S3

2. Synthesis..........S6

3. ^{1}H NMR and ^{13}C NMR Spectra..........S13

4. X-ray experimental..........S24

5. Photophysical properties..........S27

6. Metal ion sensing..........S28

7. Mechanoluminescence..........S34

8. Fluorescence of **5a**@silica..........S36

9. Supporting References..........S37

1. General Experimental

Unless otherwise stated, all reagents and solvents were obtained from commercial sources and used without further purification. Column chromatography was performed using 200-300 mesh silica gel. Compounds **1a**, **1b**, **2b**, **3b** and **9** were synthesized according to previous literature reports.[1-3]

Nuclear magnetic resonance (NMR) spectra were recorded on JEOL 400 MHz or on Bruker 600 MHz spectrometers using tetramethylsilane as the internal standard. ^{1}H NMR and ^{13}C NMR chemical shifts are reported in units of δ, parts per million (ppm), relative to the chemical shift of the residual solvent. UV-Vis spectra were recorded on a Saier 3600iPlus spectrophotometer. Fluorescence measurements were carried out using a Shimadzu RF5301 spectrofluorophotometer using a Xenon lamp as the light source. All the UV-Vis absorption and fluorescence measurements were performed in a standard 1 cm quartz cell at room temperature. Polymer films were prepared by drop-casting from 10 mg mL^{-1} chlorobenzene solutions. Powder X-ray diffraction measurements were conducted on a 18KW D/MAX2500V+/PC Powder Diffractometer with Cu-Kα radiation (λ = 0.15418 nm). Thin film X-ray diffraction (XRD) studies were carried out using a D8 discover diffractometer. Electron paramagnetic resonance (EPR) measurements were carried out using a Bruker EMX plus spectrometer. The concentration of **5a** was 5×10^{-4} mol L^{-1} and the concentration of Cu^{2+} was 10 mM, both in acetonitrile solution. Gel permeation chromatograph (GPC) measurements were carried out using a PL-GPC220 chromatograph. The polymer sample was dissolved in 1,2,4-trichlorobenzene, and stirred at 100 °C for 24 h. Before testing, the sample was pre-treated with a SP260 high temperature melting device, and the sample volume was 200 μL. GPC measurements were carried out using 1,2,4-trichorobenzene as the eluent with flow rate of 1.0 mL min^{-1} at 160 °C. Thermogravimetric analyses (TGA) were carried out on a STA449 F5 Gravimetric Thermal Analyzer using a heating rate of 10 °C min^{-1} under N_2.

UV-Vis and fluorescence spectral measurements

The concentrations of **2a**, **5a** and **8** were 10^{-5} and 10^{-6} mol L^{-1} in chloroform for UV-Vis absorption and fluorescence measurements, respectively. Dilute solutions of **DPP-C8C12** and **DPP-Cycle** in 1,2,4-trichlorobenzene were used for UV-Vis absorption and fluorescence measurements. Thin films of compounds **2a**, **5a** and **8** were prepared by spin-casting from a 10 mg mL^{-1} chloroform solution. Likewise, thin films of the two polymer samples of this study were prepared by drop-casting from 10 mg mL^{-1} 1,2,4-trichlorobenzene solutions.

Fluorescence quantum yields (Φ_F) for both solution and film measurements were measured using an integrating sphere or a standard 1 cm quartz cell. All Φ_F measurements were carried out in the air at room temperature using an Fluorolog-QM Steady State and Lifetime Fluorescence Spectrometer. The experiments were performed in duplicate.

Cu^{2+} selectivity

Stock solutions (5×10^{-3} mol L^{-1}) of the trifluorosulfonate salts of Na^{+}, Mg^{2+}, Zn^{2+}, Fe^{2+}, Ni^{2+}, K^{+}, Ca^{2+}, Ag^{+}, and Cu^{2+} in acetonitrile, and the perchlorate salt of Cd^{2+} in acetonitrile, were prepared. Test solutions were then made up by placing 3 mL the probe acetonitrile solution (1×10^{-5} mol L^{-1}) into a test tube, and then adding 100 equivalents of the Na^{+}, Mg^{2+}, Cd^{2+}, Zn^{2+}, Fe^{2+}, Ni^{2+}, K^{+}, Ca^{2+}, Ag^{+} cation salts or 5 equivalents of the corresponding Cu^{2+} salt. The mixture was allowed to equilibrate for 10 minutes before recording the fluorescence spectra. Excitation was excited at 465 nm. The excitation and emission slit widths were 1.5 and 5.0 nm, respectively.

Preparation of the 5a@Silica gel powder

Powdered silica gel (300~400 mesh, 1.0 g) was added to methanol (100 mL) and stirred gently. A chloroform solution of **5a** (1.0 mg, 0.1 wt% doping ratio) was then added. After stirring for 2 h, the supernatant became colorless, indicating that essentially all of the original luminescent molecules had been deposited on the silica. The suspension was filtered, washed with methanol, and dried at 70 °C for 2 h before use.

Fingerprint imaging

In this experiment, human fingerprints were collected from a male volunteer and deposited on various surfaces, including glass slides, copper plates, zinc plates, leather samples, transparent plastic bags, and wooden planks. Before being pressed onto the substrates, the volunteer's fingers were first cleaned using 75% ethanol wipes and then fully dried with a tissue. The volunteer then rubbed his finger on his forehead or nose tip, and gently press-stamped his fingerprint on the various substrates. The resulting fingerprint-bearing surfaces were dusted with **5a**@silica powder. Any excess powder was blown away using a rubber ear wax removal syringe. Fluorescence images of fingerprint with **5a**@ silica were illuminated using a UV lamp (365 nm) and taken using a smartphone camera.

2. Synthesis

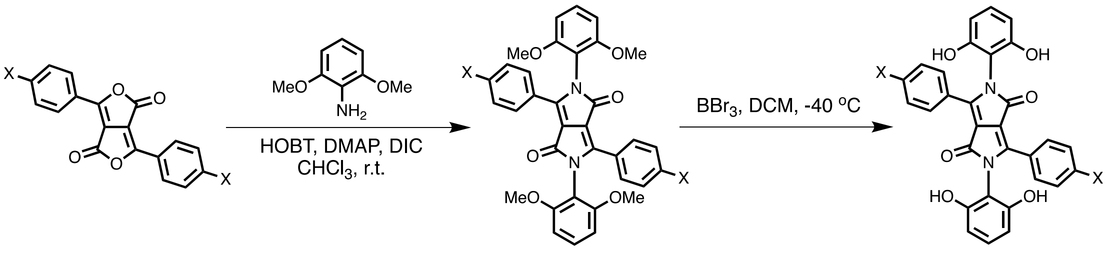

1a: X = H
1b: X = Br

2a: X = H, 21%
2b: X = Br, 16%

3a: X = H, 93%
3b: X = Br, 95%

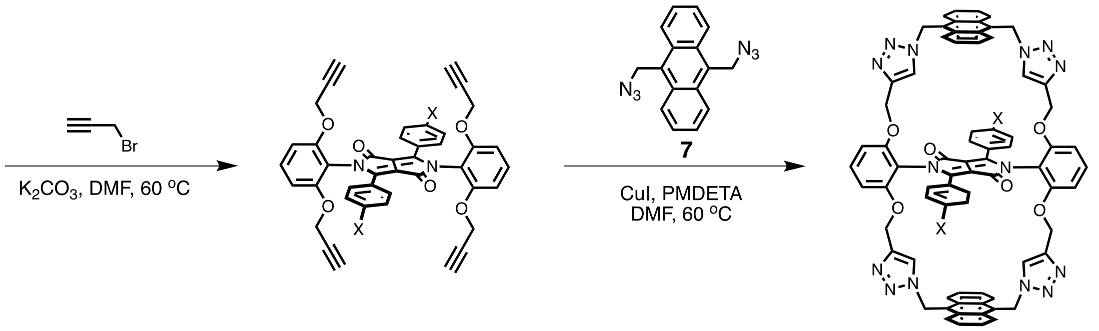

4a: X = H, 62%
4b: X = Br, 49%

5a: X = H, 9%
5b: X = Br, 4%

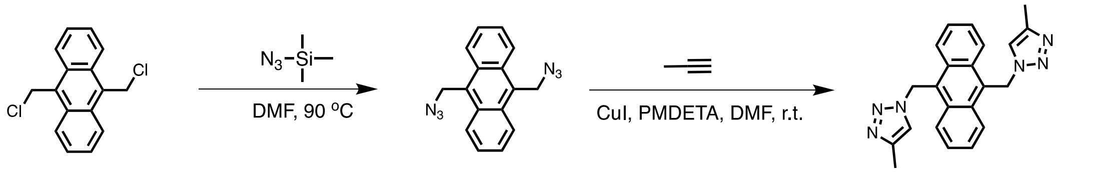

6

7, 46%

8, 35%

2,5-Bis(2,6-dimethoxyphenyl)-3,6-diphenyl-2,5-dihydropyrrolo[3,4-c]pyrrole-1,4-dione (2a)

A mixture of compound **1a** (3 g, 10.32 mmol), 2,6-dimethoxyaniline (6.33 g, 41.28 mmol), 1-hydroxybenzotriazole hydrate (5.66 g, 41.28 mmol), 4-(dimethylamino)pyridine (5.04 g, 41.28 mmol), *N*, *N*'-diisopropylcarbodiimide (6.5 mL, 41.28 mmol) and chloroform (250 mL) were stirred at RT for 7 d. The resulting mixture was then concentrated in vacuo. Methanol was added and the precipitate that formed was isolated by filtration to afford crude product, which was further purified by silica gel column chromatography using ethyl acetate/dichloromethane (from 1:50 to 2:50, v/v) as the eluent to give an orange powder (1.2 g, yield: 21%).

^{1}H NMR (400 MHz, $CDCl_3$) δ (ppm): 7.69 (d, *J* = 7.1, 4H), 7.33 (d, *J* = 7.1, 4H), 7.28-7.26 (m, 2H), 7.23 (d, *J* = 8.5 Hz, 2H), 6.58 (d, *J* = 8.5 Hz, 4H), 3.68 (s, 12H). ^{13}C NMR (100 MHz, $CDCl_3$) δ (ppm): 162.1, 157.1, 148.4, 130.5, 130.2, 128.8, 128.5, 128.1, 114.0, 110.2, 104.5, 56.1.

2,5-Bis(2,6-dihydroxyphenyl)-3,6-diphenyl-2,5-dihydropyrrolo[3,4-c]pyrrole-1,4-dione (3a)

A solution of **2a** (0.3 g, 0.54 mmol) in anhydrous dichloromethane (50 mL) was cooled to -40 °C. Boron tribromide solution (8.6 mL, 16 equiv., 1 M in dichloromethane) was then added slowly to the solution. The resulting mixture was allowed to warm to RT and stirred overnight. Methanol was slowly added to quench the reaction. The resulting solution was concentrated under reduced pressure. The crude product obtained in this way was sonicated in hexanes, and the solid was filtered to afford the title product as an orange powder (0.25 g, yield: 93%). This material was used in the next synthetic step without further purification.

^{1}H NMR (400 MHz, DMSO-d_6) δ (ppm): 9.70 (s, 4H), 7.68 (d, *J* = 8.3, 4H), 7.36–7.27 (m, 6H), 6.98 (t, *J* = 8.2 Hz, 2H), 6.33 (d, *J* = 8.2 Hz, 4H). ^{13}C NMR (100 MHz, DMSO-$d_{6)}$ δ 162.0, 156.0, 148.2, 131.4, 130.4, 128.8, 128.7, 128.6, 111.8, 110.0, 107.0.

2,5-Bis(2,6-bis(prop-2-yn-1-yloxy)phenyl)-3,6-diphenyl-2,5-dihydropyrrolo[3,4-c]pyrrole-1,4-dione (4a)

A solution of **3a** (0.3 g, 0.59 mmol), potassium carbonate (0.8 g, 5.9 mmol) and 3-bromopropyne (0.7 g, 0.5 mL, 5.9 mmol) in anhydrous DMF (15 mL) was heated to 60 °C under nitrogen for 12 h. Water was added to quench the reaction, and the mixture was extracted with dichloromethane. The organic layer was washed with brine and dried over Na_2SO_4. The solvent was removed under reduced pressure. The crude product obtained in this way was purified by silica gel column chromatography using dichloromethane/ethyl acetate (1:99, v/v) as the eluent to afford an orange powder (0.24

g, yield: 62%).

^{1}H NMR (400 MHz, DMSO-d_6) δ (ppm): 7.55 (d, J = 7.2 Hz, 4H), 7.43–7.33 (m, 4H), 7.33–7.24 (m, 4H), 6.84 (d, J = 8.6 Hz, 4H), 4.73 (d, J = 2.4 Hz, 8H), 3.46 (t, J = 2.4, 4H). ^{13}C NMR (100 MHz, DMSO-d_6) δ (ppm): 161.2, 155.1, 148.2, 131.8, 130.8, 128.7, 128.5, 128.0, 114.0, 109.4, 106.9, 79.2, 79.0, 56.4. Elemental Analysis for $C_{42}H_{28}N_2O_6$: C, 76.82; H, 4.30; N, 4.27; O, 14.62. Found: C, 76.88; H, 4.27; N, 4.18; O, 14.67. MS (MALDI-TOF): Calcd for $C_{42}H_{29}N_2O_6$ [M+H]$^+$: 657.2019, found: 657.2020; Calcd for $C_{42}H_{28}N_2O_6Na$ [M+Na]$^+$: 679.1845, found: 679.1849.

2,5-bis(2,6-bis(prop-2-yn-1-yloxy)phenyl)-3,6-bis(4-bromophenyl)-2,5-dihydropyrrolo[3,4-c]pyrrole-1,4-dione (4b)

Following the procedure used for the preparation of compound **4a**, this compound was prepared from compound **3b** (0.17 g, 0.26 mmol), potassium carbonate (0.4 g, 2.6 mmol), and 3-bromopropyne (0.3 g, 0.15 mL, 2.6 mmol). After purification by silica gel column chromatography using dichloromethane as the eluent, the title compound was isolated as a brown solid (0.10 g, yield: 49%).

^{1}H NMR (400 MHz, DMSO-d_6) δ (ppm): 7.53 (d, J = 8.8 Hz, 4H), 7.46 (d, J = 8.8 Hz, 4H), 7.41 (t, J = 8.7 Hz, 2H), 6.85 (d, J = 8.6 Hz, 4H), 4.76 (s, 8H), 3.48 (t, J = 2.3 Hz, 4H). ^{13}C NMR (100 MHz, DMSO-d_6) δ (ppm): 161.0, 155.0, 147.2, 131.9, 131.0, 130.3, 127.0, 125.6, 113.6, 109.6, 107.1, 79.2, 79.0, 56.4. Elemental Analysis for $C_{42}H_{26}Br_2N_2O_6$: C, 61.94; H, 3.22; Br, 19.62; N, 3.44; O, 11.79. Found: C, 61.99; H, 3.20; Br, 19.57; N, 3.47; O, 11.77. MS (MALDI-TOF): Calcd for $C_{42}H_{30}N_2O_6$ [M+H]$^+$: 813.0236, found: 813.0211; Calcd for $C_{42}H_{29}N_2O_6Na$ [M+Na]$^+$: 835.0056, found: 835.0057.

Macrocycle 5a

Compound **4a** (200.0 mg, 0.305 mmol) and compound **7** (176 mg, 0.61 mmol) were

added to DMF (100 mL). The solution was bubbled with nitrogen for 10 minutes to purge it largely from ambient air. Following this, CuI (34.8 mg, 0.183 mmol) and *N,N,N,N*-pentamethyldiethylenetriamine (PMDETA) (38.2 μL, 31.7 mg, 0.183 mmol) were added to the solution. The mixture was then heated to 60 °C and held there for 24 h. The mixture was allowed to cool to room temperature before being quenched by water. The resulting solution was extracted with dichloromethane and the organic phase dried over Na_2SO_4. The solvent was removed under reduced pressure. The crude product obtained in this way was purified by silica gel column chromatography using methanol/dichloromethane (2:98, v/v) as the eluent to afford a yellow solid (25 mg, yield: 9%).

^{1}H NMR (600 MHz, DMSO-d_6) δ (ppm): 8.65 (s, 8H), 7.76 (s, 4H), 7.59 (s, 8H), 7.39 (d, J =16.0 Hz, 4H), 7.22 (d, J = 7.7 Hz, 4H), 7.08 (d, J = 8.1 Hz, 4H), 6.95 (d, J = 7.7 Hz, 4H), 6.68 (s, 8H), 4.63 (d, J = 14.1 Hz, 8H). ^{13}C NMR (150 MHz, DMSO-d_6) δ (ppm): 160.0, 155.5, 147.4, 142.6, 131.2, 130.4, 130.2, 128.8, 127.9, 127.6, 127.4, 125.5, 125.1, 113.7, 108.8, 107.3, 61.7, 45.6. Elemental Analysis for $C_{74}H_{52}N_{14}O_6$: C, 72.07; H, 4.25; N, 15.90; O, 7.78. Found: C, 71.99; H, 4.27; N, 16.12; O, 7.62. MS (MALDI-TOF): Calcd for $C_{74}H_{53}N_{14}O_6$ $[M+H]^+$: 1233.42, found: 1233.23; Calcd for $C_{74}H_{52}N_{14}O_6Na$ $[M+Na]^+$: 1255.41, found: 1256.24.

Macrocycle 5b

Following the procedure used for the preparation of compound **5a**, this compound was prepared from compound **4b** (248.0 mg, 0.305 mmol), compound **7** (176.0 mg, 0.61 mmol), CuI (34.8 mg, 0.183 mmol) and PMDETA (38.2 μL, 31.7 mg, 0.183 mmol). Purification by silica gel column chromatography using methanol/dichloromethane (1:99, v/v) as the eluent gave a yellow solid (18 mg, yield: 4%).

^{1}H NMR (400 MHz, DMSO-d_6) δ (ppm): 8.65 (s, 8H), 7.72 (s, 4H), 7.56 (s, 8H), 7.38 (d, J = 8.4 Hz, 6H), 7.06 (d, J = 8.5 Hz, 4H), 6.84 (d, J = 8.7 Hz, 4H), 6.65 (s, 8H), 4.62 (s, 8H). ^{13}C NMR (100 MHz, DMSO-d_6) δ 159.9, 155.5, 146.5, 142.7, 131.6, 130.8, 130.3, 129.7, 128.9, 127.5, 126.7, 125.5, 125.0, 113.3, 109.2, 107.6, 61.9, 45.7.

Elemental Analysis for $C_{74}H_{50}Br_2N_{14}O_6$: C, 63.89; H, 3.62; Br, 11.49; N, 14.10; O, 6.90 Found: C, 63.80; H, 3.52; Br, 11.63; N, 14.17; O, 6.88. MS (MALDI-TOF): Calcd for $C_{74}H_{50}N_{14}O_6Br_2Na$ $[M+Na]^+$: 1413.24, found: 1413.48.

9,10-Bis(azidomethyl)anthracene (7)

9,10-Bis(chloromethyl)anthracene (1 g, 3.6 mmol) and azidotrimethylsilane (2 mL, 15.2 mmol) were dissolved in DMF (10 mL). This reaction mixture was heated to 90 °C and left to stir overnight at this temperature. The reaction mixture was allowed to cool to room temperature, and then quenched with water. The resulting precipitate was collected by filtration and washed first with water and then n-hexane to yield a crude product. This crude product was purified by silica gel column chromatography using dichloromethane/petroleum ether (from 1:4 to 3:7, v/v) as the eluent to afford a green powder (0.48 g, yield: 46%).

^1H NMR (400 MHz, $CDCl_3$) δ (ppm): 8.41–8.32 (m, 4H), 7.68– 7.59 (m, 4H), 5.36 (s, 4H). ^{13}C NMR (100 MHz, $CDCl_3$) δ (ppm): 130.5, 128.2, 126.7, 124.5, 46.5.

Compound 8

Following the procedure used for the preparation of compound **5a** and **5b**, this compound was prepared using propyne (0.92 mL, 1 mol/L in *N,N*-dimethylformamide, 0.92 mmol), compound **7** (133.0 mg, 0.46 mmol), CuI (34.8 mg, 0.183 mmol) and PMDETA (38.2 μL, 31.7 mg, 0.183 mmol). Purification by silica gel column chromatography using methanol/dichloromethane (2:98, v/v) as the eluent to give a green solid (60 mg, yield: 35%). Because of the poor solubility of compound **8**, the carbon spectrum was not recorded.

^1H NMR (400 MHz, $CDCl_3$) δ (ppm): 8.45 (d, J = 10.1 Hz, 4H), 7.66 (d, J = 10.1 Hz, 4H), 6.91 (s, 2H), 6.54 (s, 4H), 2.18 (s, 6H). MS (MALDI-TOF): Calcd for $C_{22}H_{22}N_6Na$ $[M+Na]^+$: 391.17, found: 391.15.

Monomer 10

3,6-Bis(4-bromophenyl)pyrrolo[3,4-c]pyrrole-1,4(2H,5H)-dione (1.0 g, 2.24 mmol), cesium carbonate (1.46 g, 4.48 mmol) and 9-(bromomethyl)nonadecane (2.43 g, 6.72 mmol) in anhydrous DMF (30 mL) was heated to 100 °C under nitrogen and held at this temperature for 12 h. After allowing to cool to room temperature, the mixture was extracted with dichloromethane and water. The organic layer was washed with brine and dried over Na_2SO_4. The volatiles were removed from the dried organic phase under reduced pressure. The crude product obtained in this way was purified by silica gel column chromatography using dichloromethane/ethyl acetate (1:1, v/v) as the eluent to afford an orange powder (0.55 g, yield: 48.9%). ^1H NMR (400 MHz, $CDCl_3$) δ (ppm): 7.62 (s, 8H), 3.68 (d, J = 7.3 Hz, 4H), 1.49 (s, 2H), 1.25-1.20 (m, 42H), 1.09–1.01 (m, 22H), 0.91–0.82 (m, 12H). ^{13}C NMR (101 MHz, $CDCl_3$) δ (ppm): 162.6, 147.7, 132.2, 130.1, 127.4, 125.7, 109.9, 45.3, 37.1, 32.0, 31.3, 29.9, 29.7, 29.5, 26.4, 26.1, 22.8, 21.1, 14.2.

Synthesis of polymer

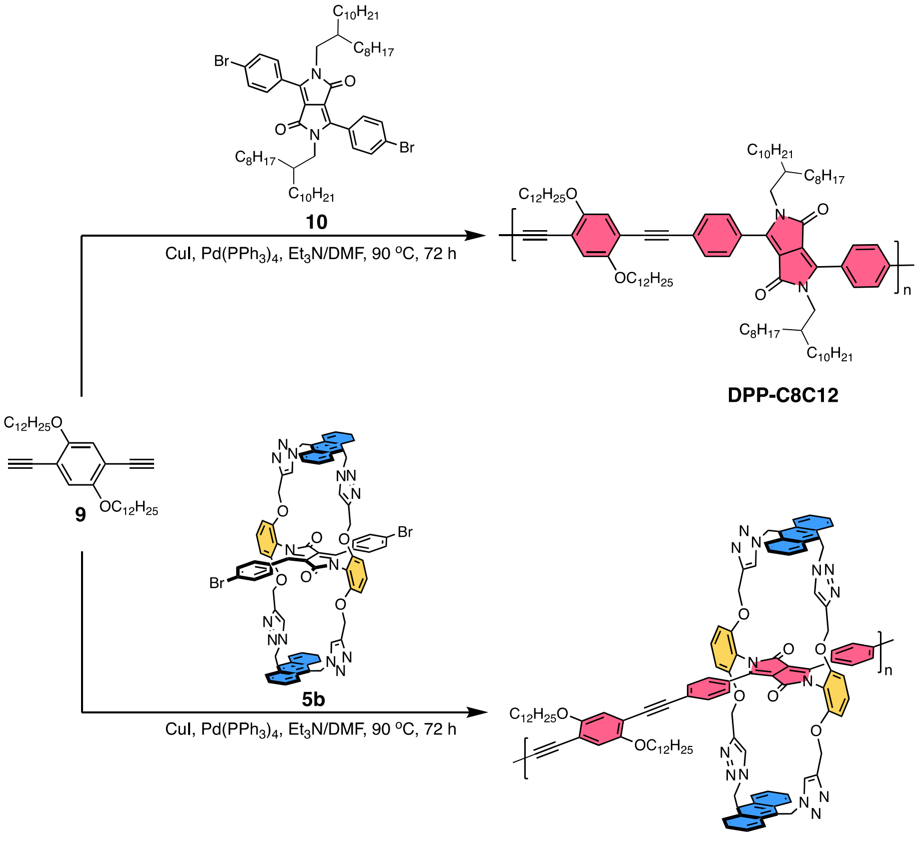

Polymer DPP-C8C12

Monomers **9** (48.98 mg, 0.099 mmol) and **10** (100.0 mg, 0.099 mmol), tetrakis-(triphenylphosphine) palladium (11.44 mg, 0.0099 mmol), and CuI (3.77 mg, 0.0198 mmol) were placed in a 50 mL Schlenk flask under a protective nitrogen atmosphere, before DMF/triethylamine (10 mL, v/v = 1:1) were added. The flask was then degassed through three vacuum-nitrogen cycles. The resulting reaction mixture was stirred at 90 ^{o}C for three days. After allowing to cool to room temperature, methanol was slowly added to precipitate the solid, which was collected by filtration. This solid was purified by successive Soxhlet extractions using in sequence methanol, acetone, hexane and chloroform. The residual mass was dried in a vacuum oven for 24 h at 60 ^{o}C to give **DPP-C8C12** as a red solid (88.6 mg, yield: 66.6%). M_n = 17.7 kg mol^{-1}, M_w = 35.7 kg mol^{-1}, DPI = 2.02.

Polymer DPP-Cycle

Following the procedure used for the preparation of polymer **DPP-C8C12**, polymer **DPP-Cycle** was prepared using monomers **9** (17.81 mg, 0.036 mmol) and **5b** (50.0 mg, 0.036 mmol), CuI (1.37 mg, 0.0072 mmol), and tetrakis-(triphenylphosphine) palladium (4.16 mg, 0.0036 mmol). After allowing to cool room temperature, then methanol was slowly added to precipitate the solid, which was collected by filtration. This solid was purified by successive Soxhlet extractions using in sequence methanol, acetone, hexane and chloroform. The residual mass was dried in a vacuum oven for 24 h at 60 ^{o}C to give **DPP-Cycle** as a red solid (43.0 mg, yield: 69.0%). M_n = 25.5 kg mol^{-1}, M_w = 37.1 kg mol^{-1}, DPI = 1.45.

3. ¹H NMR and ¹³C NMR Spectra

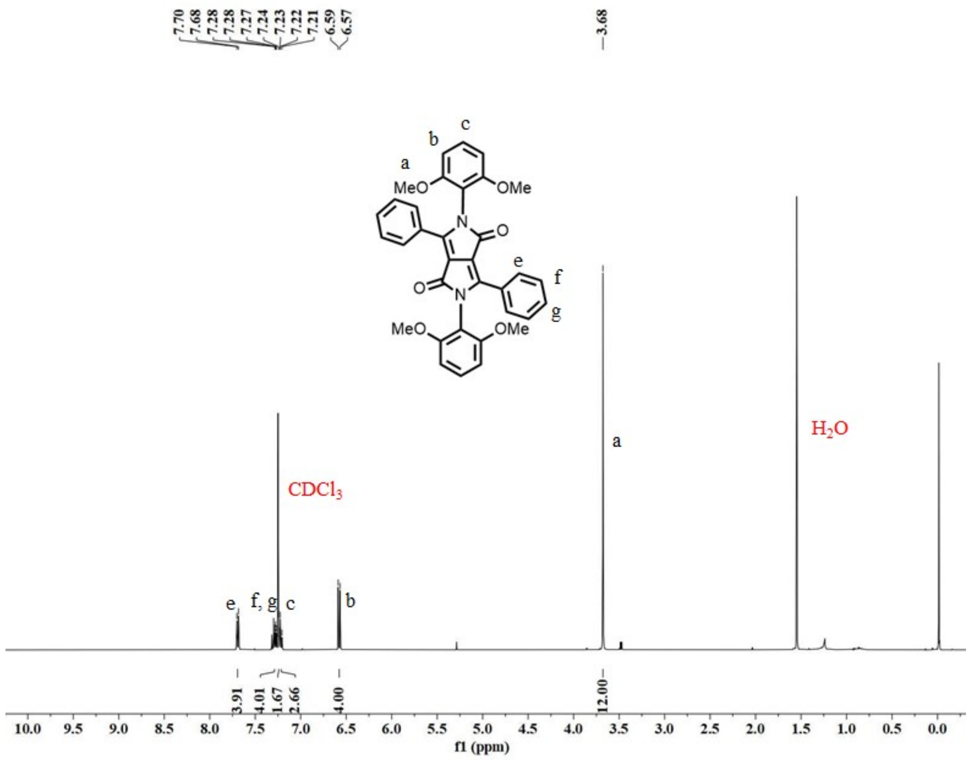

Figure S1. ¹H NMR spectrum of compound **2a** in $CDCl_3$.

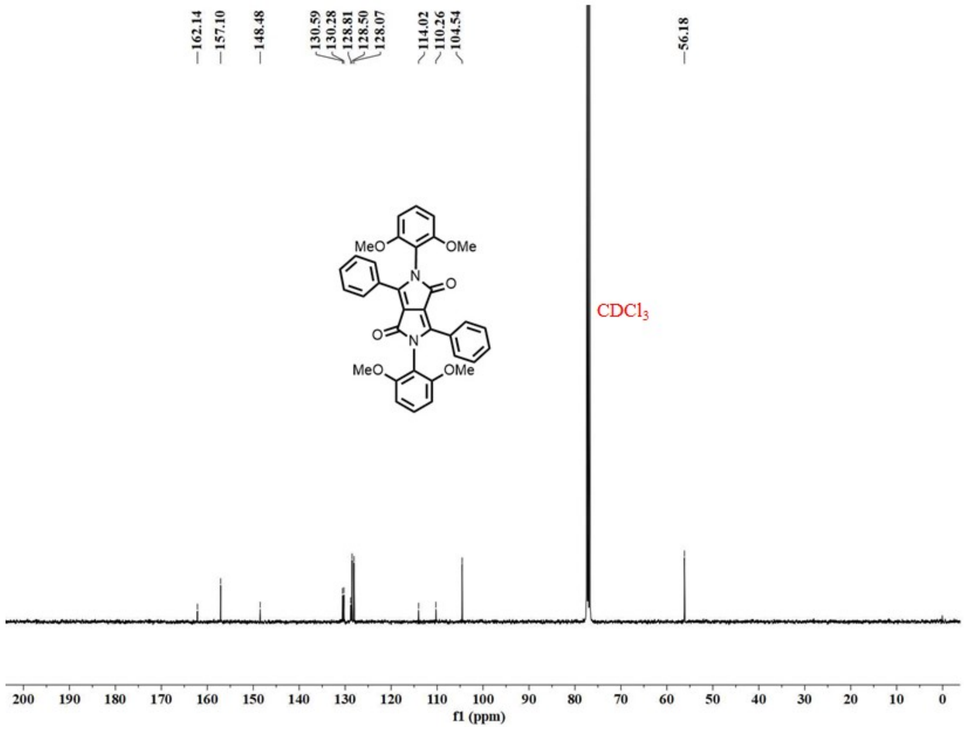

Figure S2. ¹³C NMR spectrum of compound **2a** in $CDCl_3$.

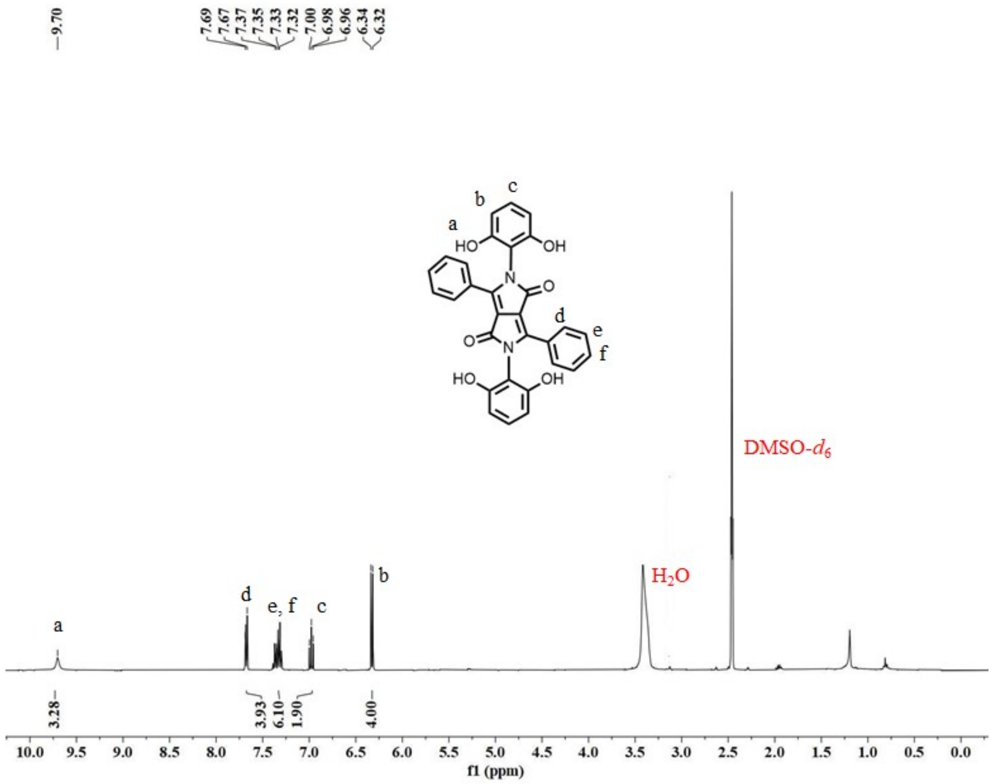

Figure S3. ^{1}H NMR spectrum of compound **3a** in DMSO-d_6.

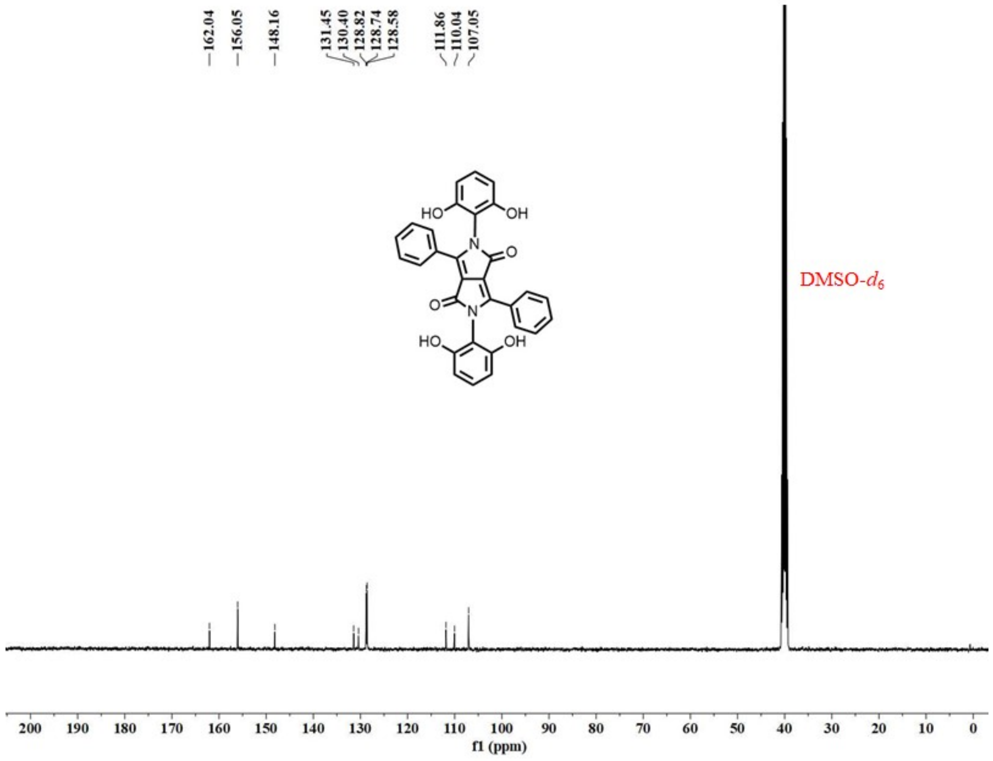

Figure S4. ^{13}C NMR spectrum of compound **3a** in DMSO-d_6.

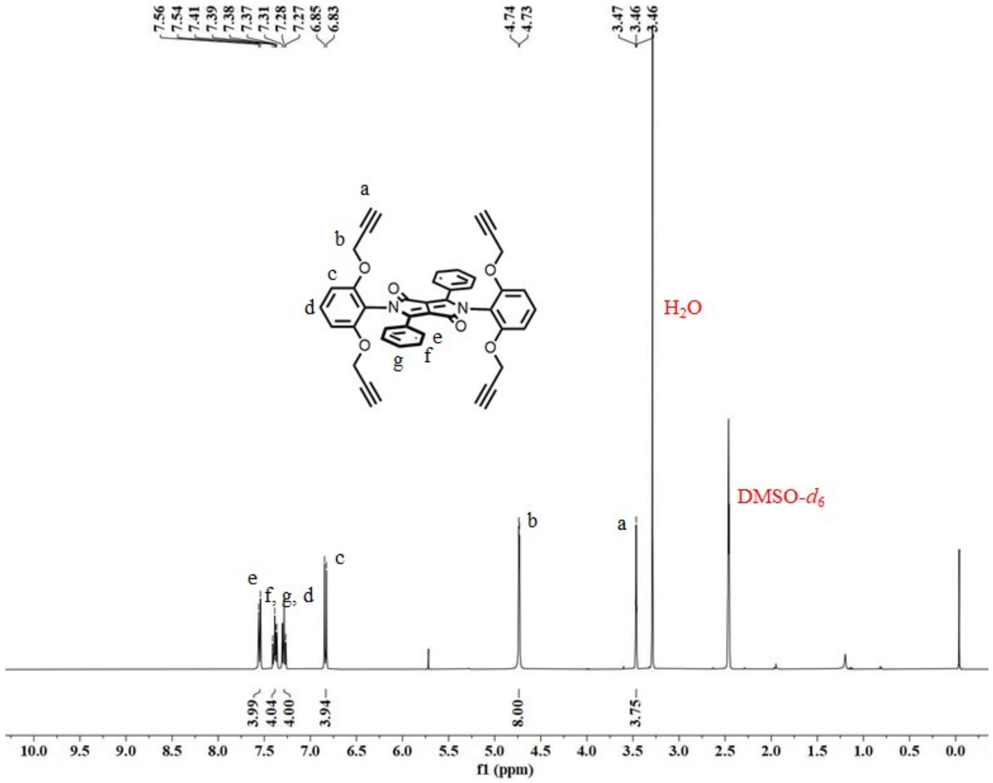

Figure S5. ^{1}H NMR spectrum of compound **4a** in DMSO-d_6.

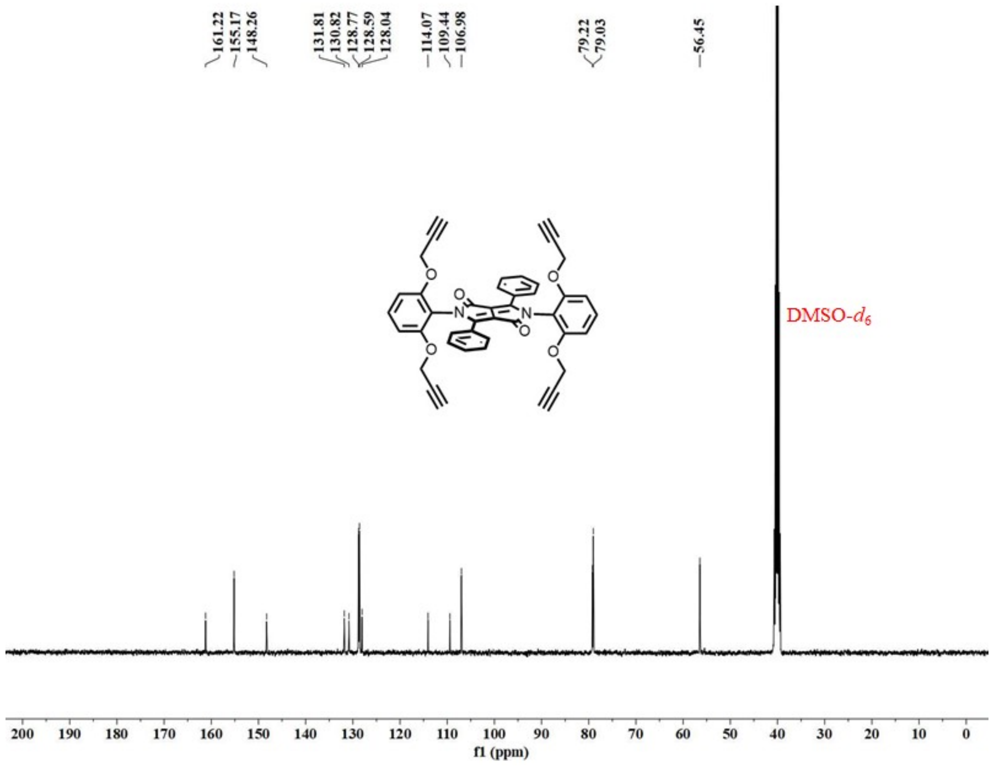

Figure S6. ^{13}C NMR spectrum of compound **4a** in DMSO-d_6.

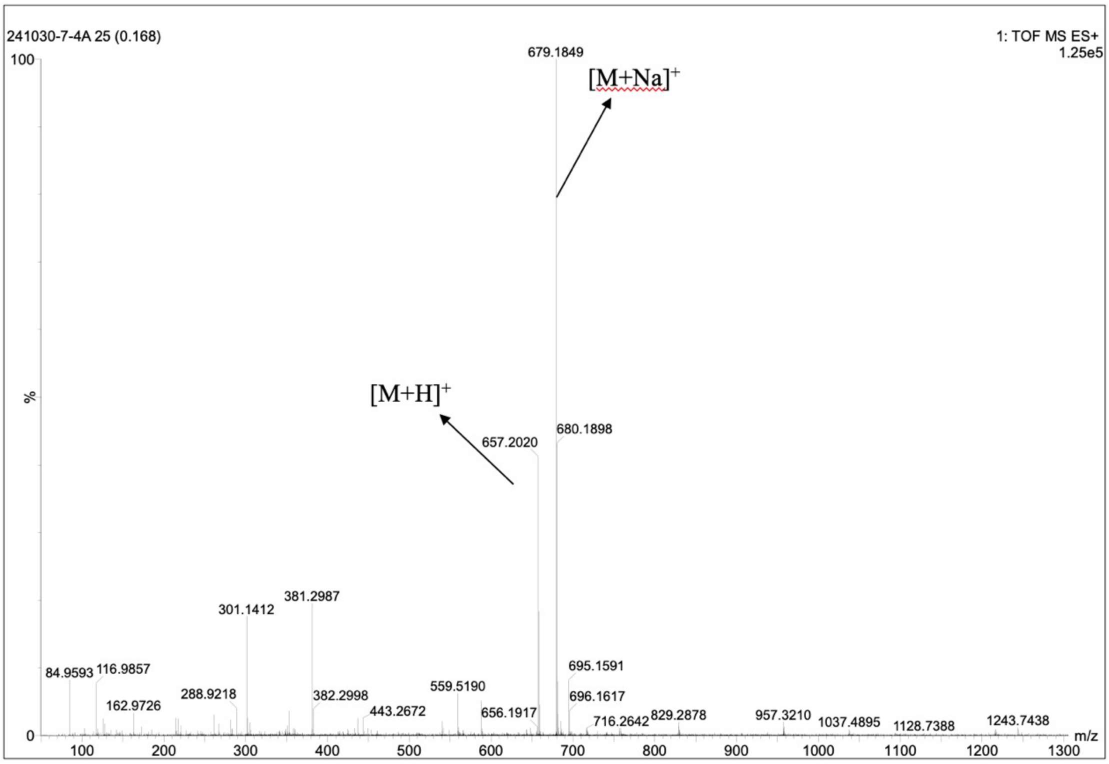

Figure S7. ESI-TOF Mass spectrum of compound **4a**.

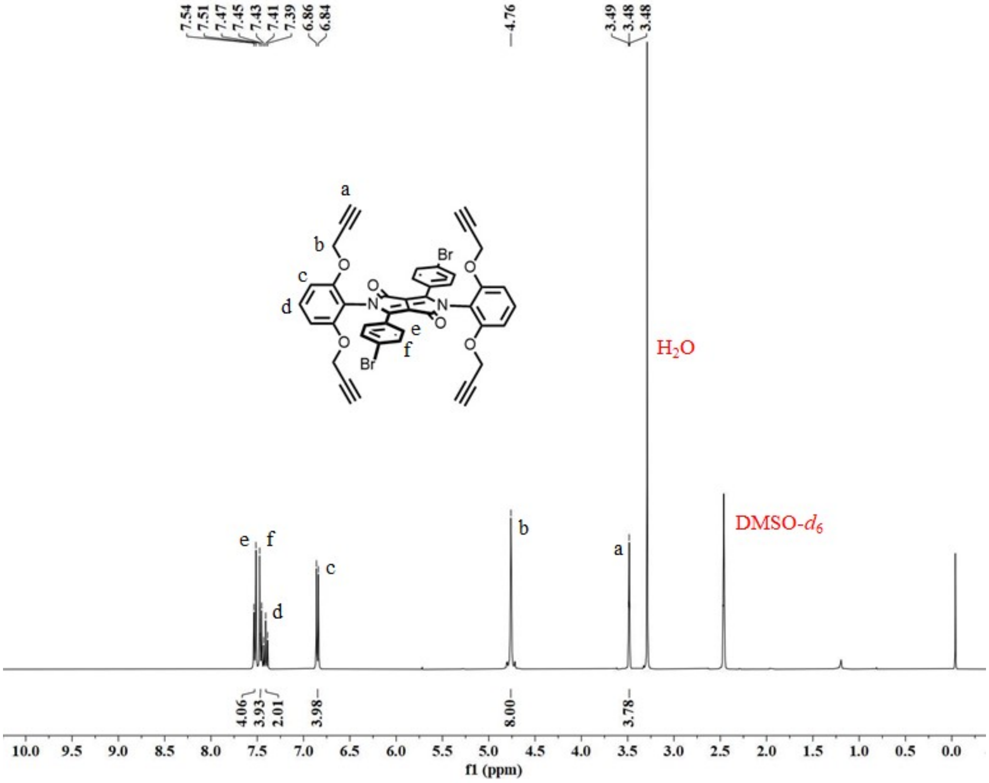

Figure S8. ¹HNMR spectrum of compound **4b** in DMSO-d_6.

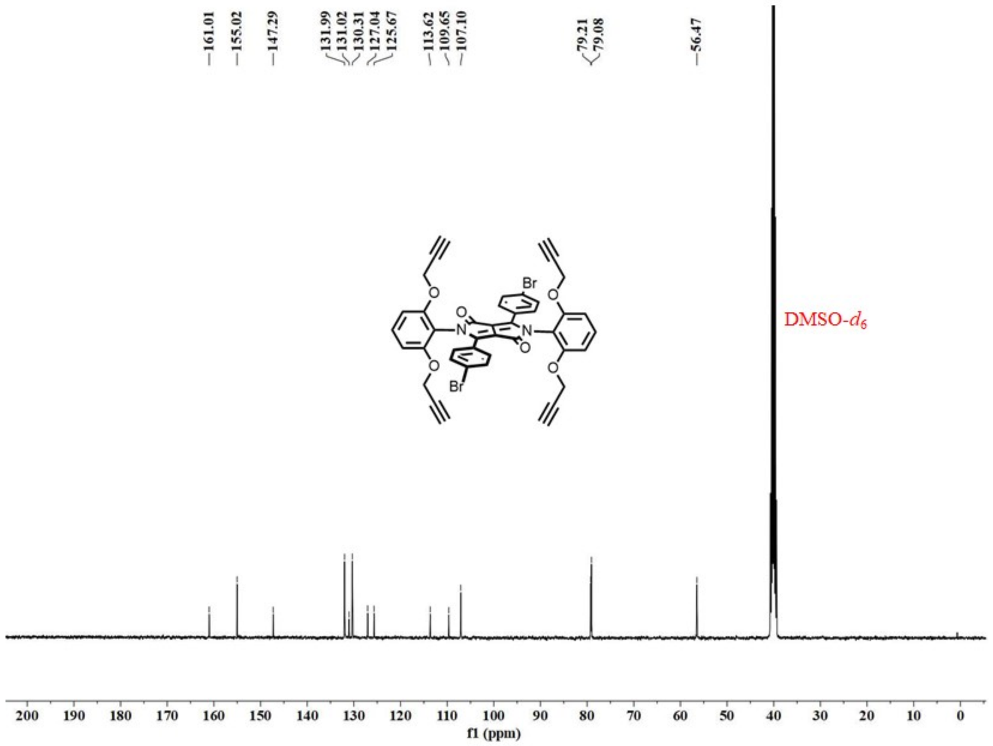

Figure S9. ^{13}C NMR spectrum of compound **4b** in DMSO-d_6.

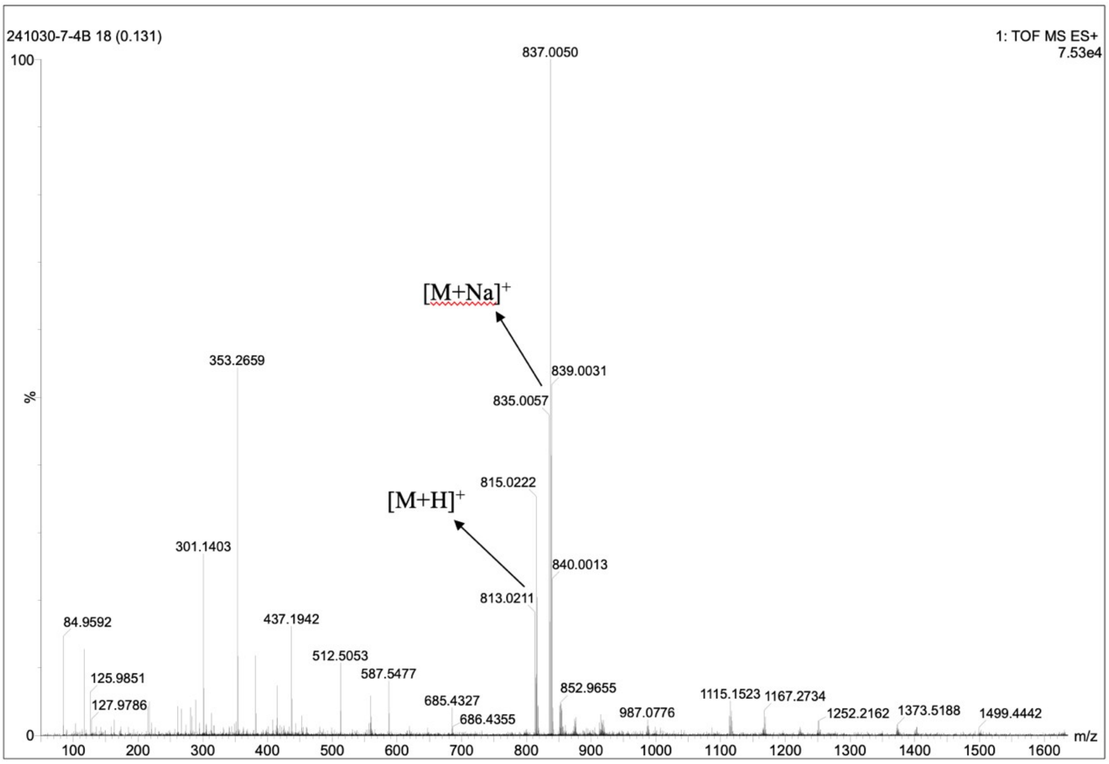

Figure S10. ESI-TOF Mass spectrum of compound **4b**.

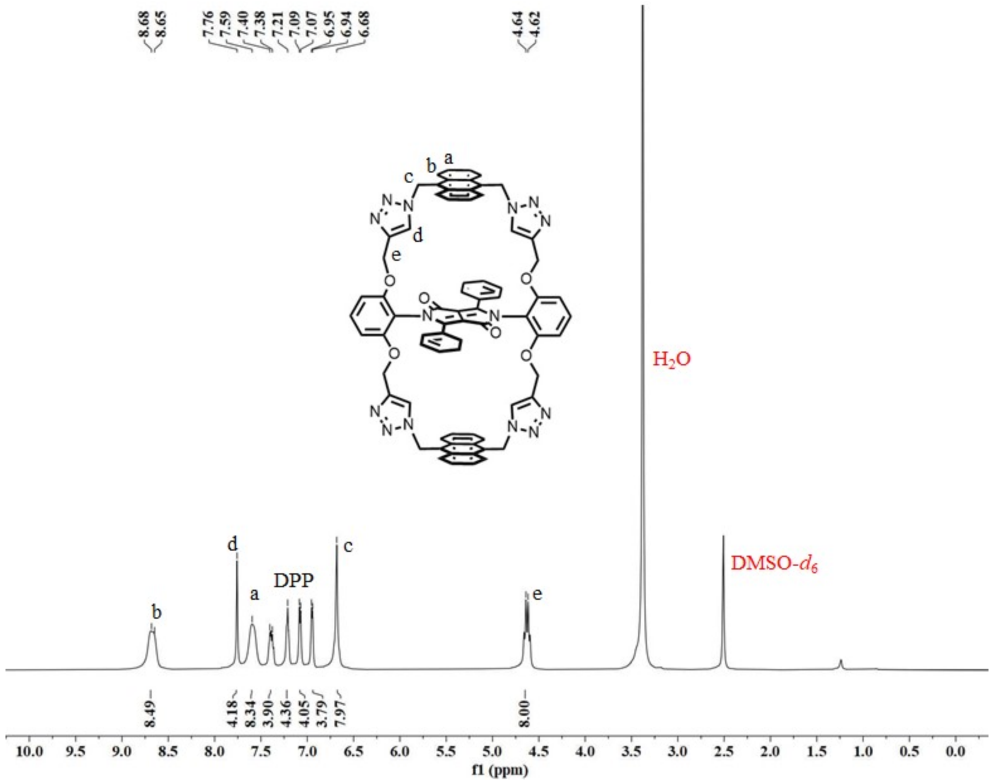

Figure S11. ^{1}H NMR spectrum of compound **5a** in DMSO-d_6.

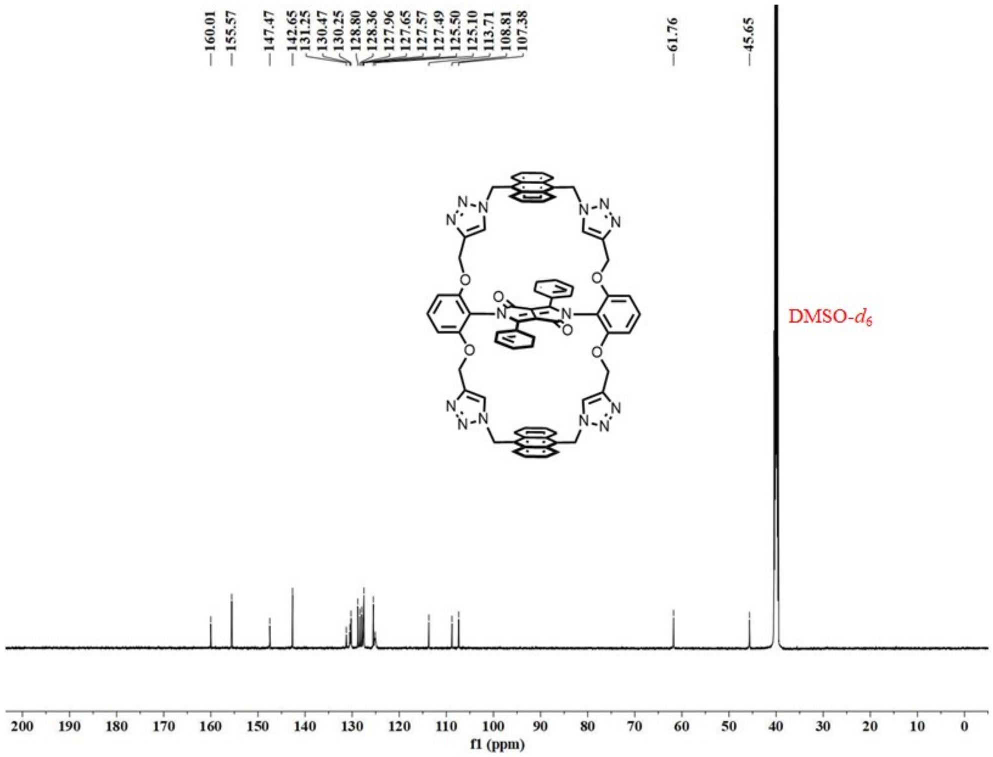

Figure S12. ^{13}C NMR spectrum of compound **5a** in DMSO-d_6.

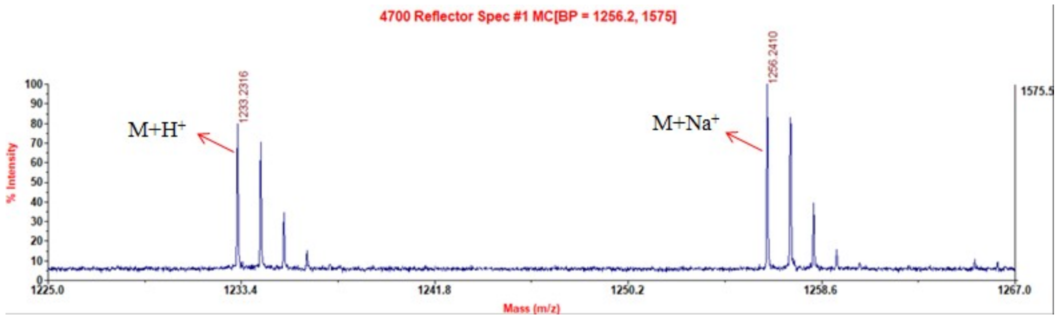

Figure S13. MALDI-TOF MS spectrum of compound **5a**.

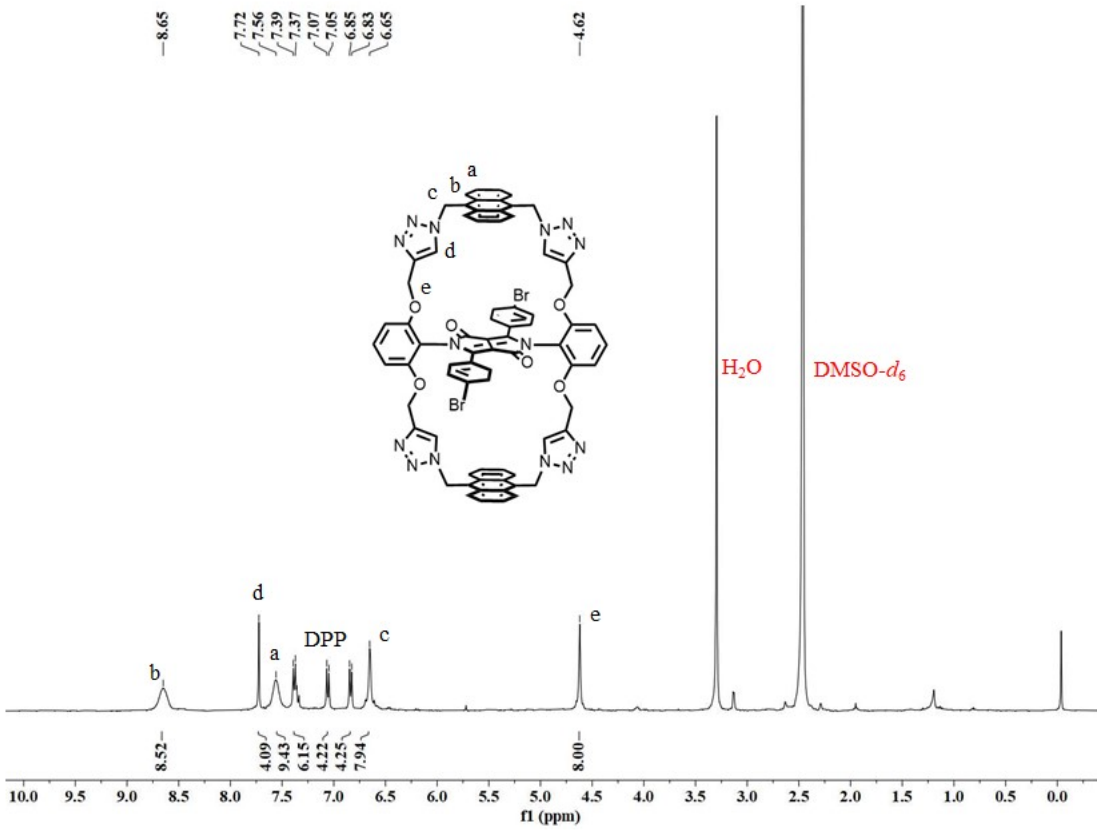

Figure S14. ^{1}H NMR spectrum of compound **5b** in DMSO-d_6.

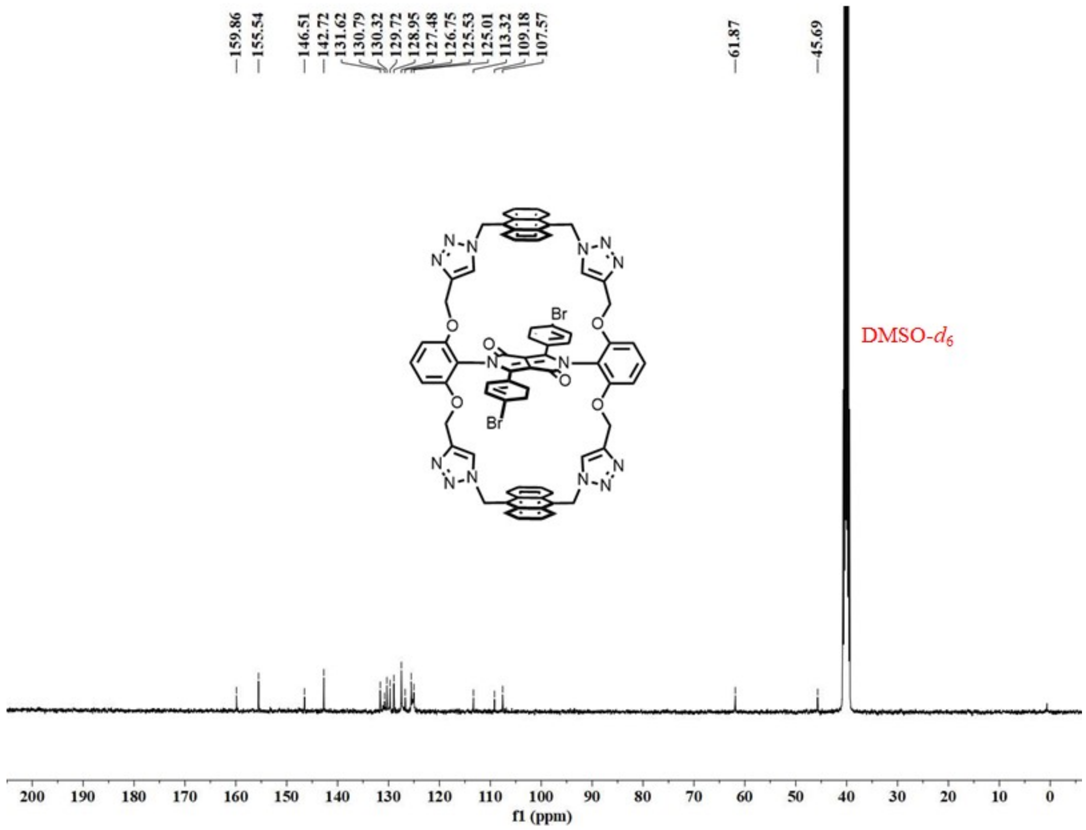

Figure S15. ^{13}C NMR spectrum of compound **5b** in DMSO-d_6.

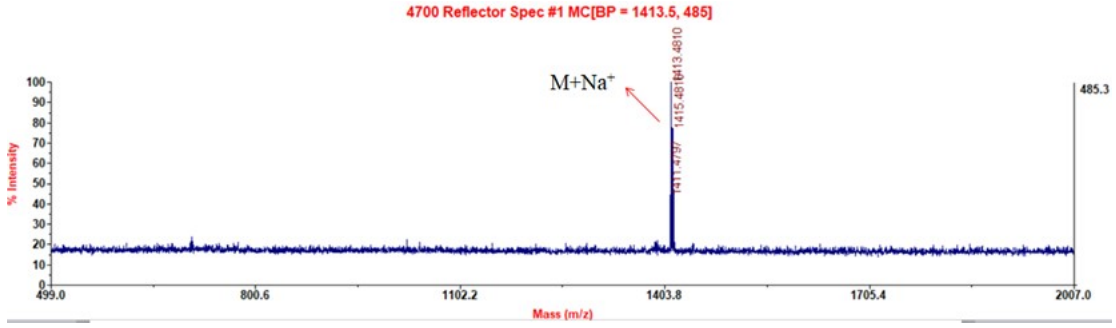

Figure S16. MALDI-TOF MS spectrum of compound **5b**.

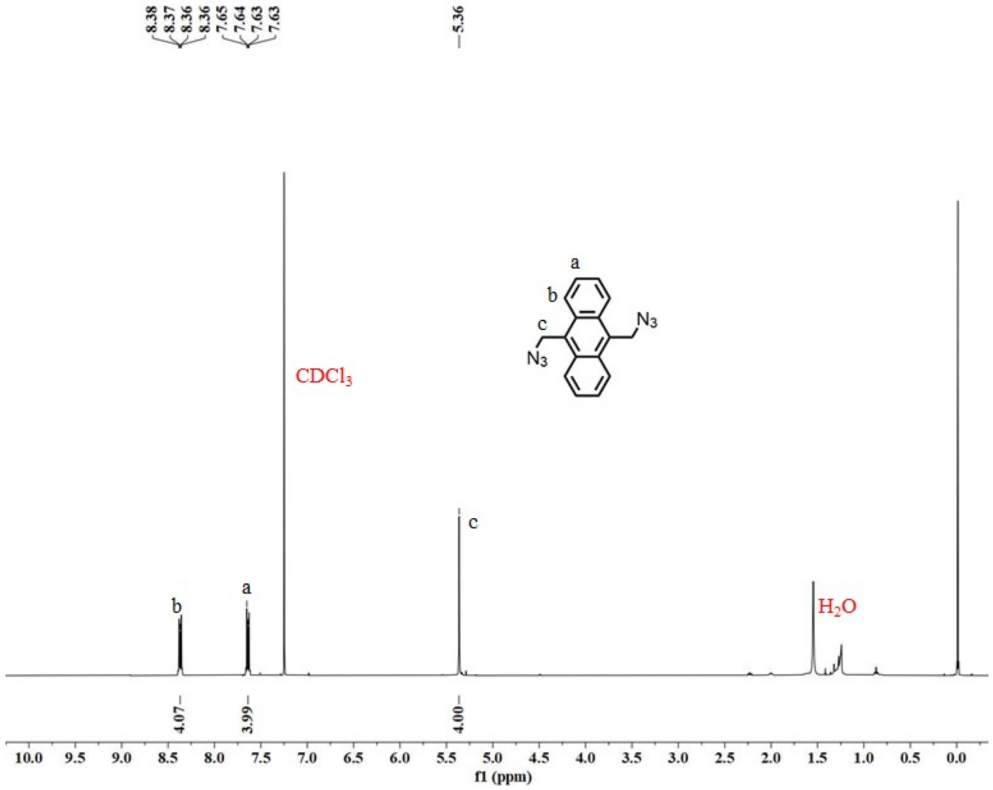

Figure S17. ^{1}H NMR spectrum of compound **7** in $CHCl_3$.

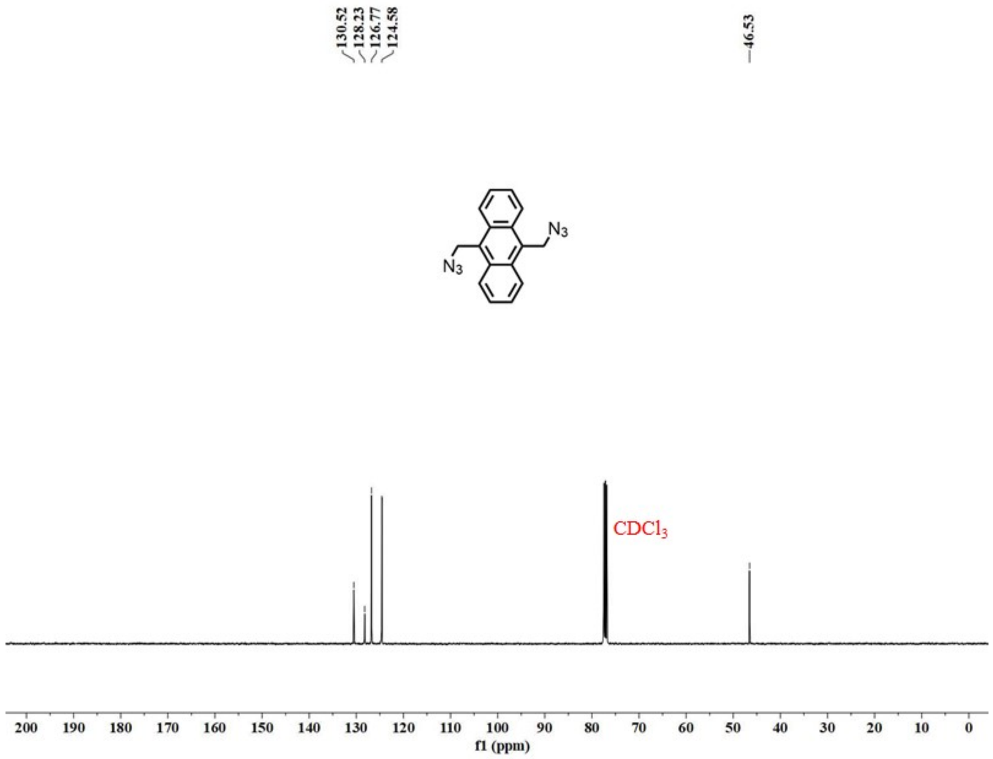

Figure S18. ^{13}C NMR spectrum of compound **7** in $CHCl_3$.

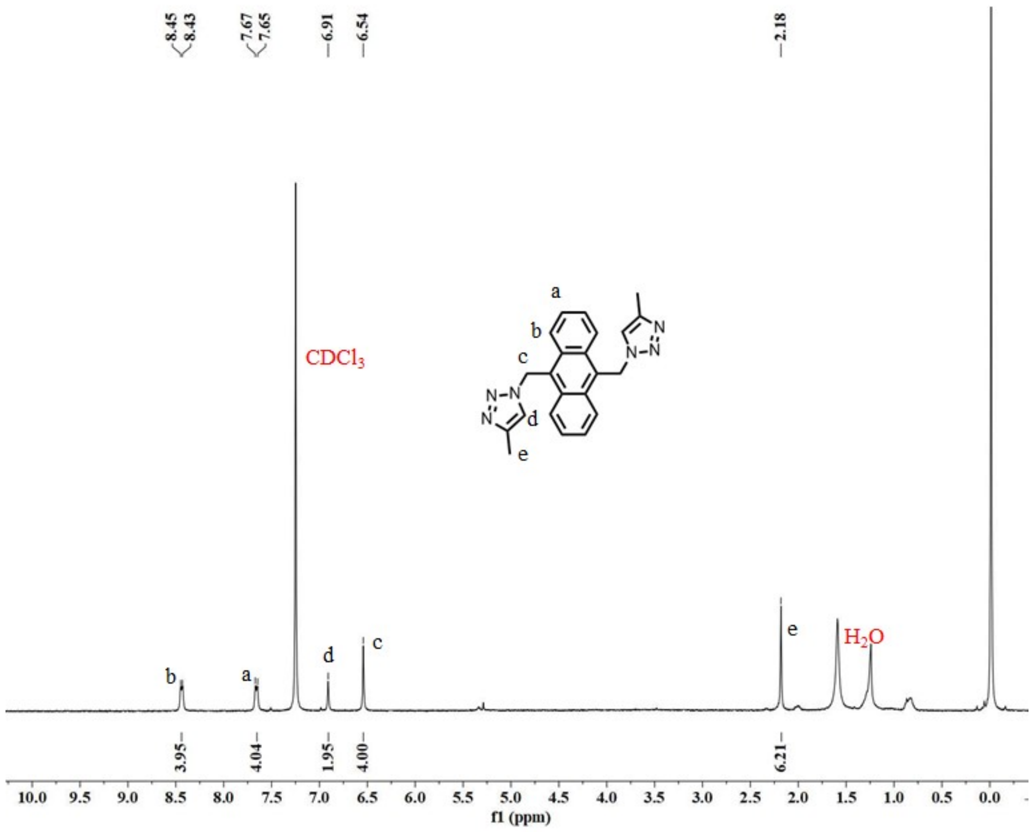

Figure S19. ^{1}H NMR spectrum of compound **8** in $CDCl_3$. Due to its poor solubility in common NMR solvents, the ^{13}C NMR spectrum of compound **8** could not be recorded.

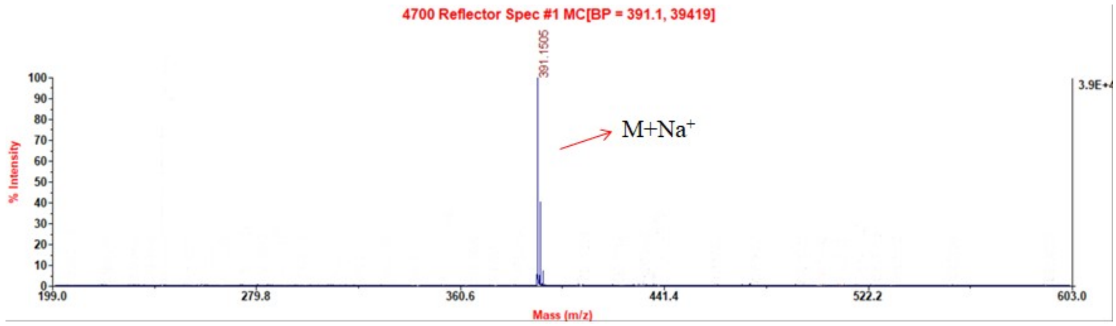

Figure S20. MALDI-TOF MS spectrum of compound **8**.

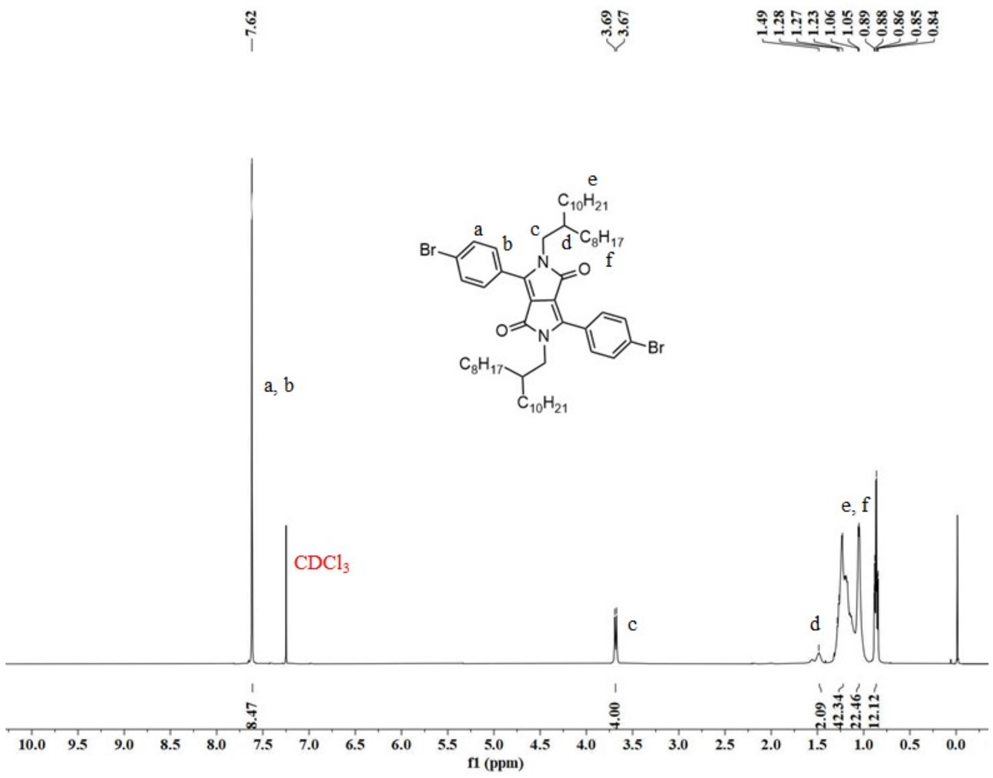

Figure S21. ^{1}H NMR spectrum of monomer **10** in $CDCl_3$.

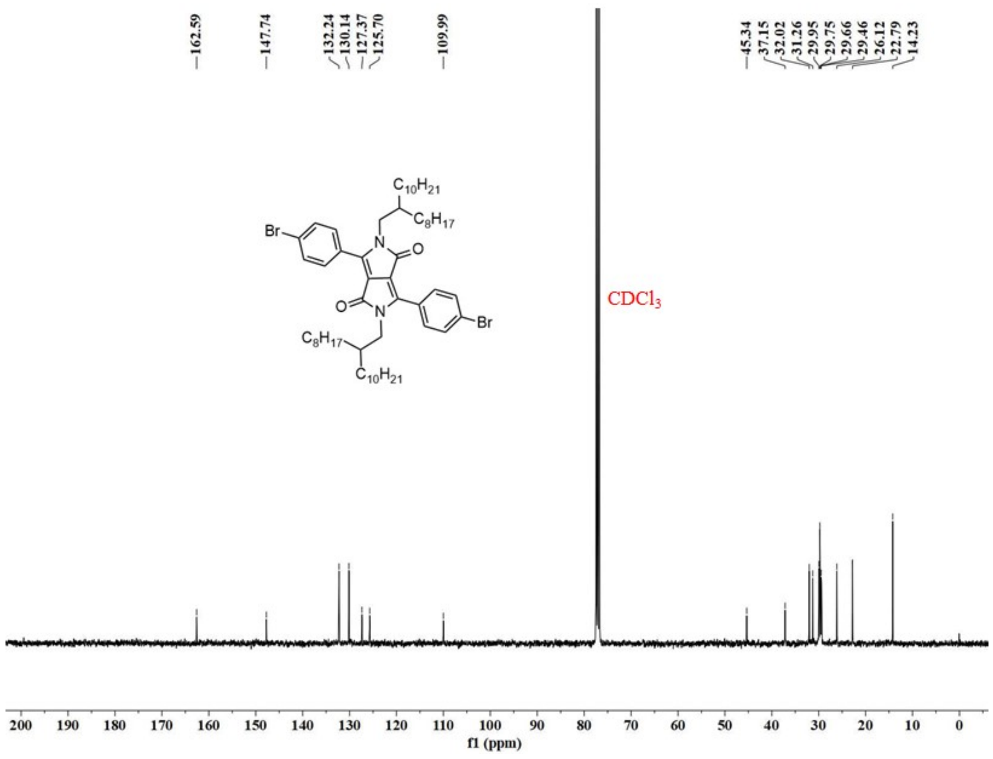

Figure S22. ^{13}C NMR spectrum of monomer **10** in $CDCl_3$.

4. X-ray experimental

Diffraction grade single crystals of **2a** were obtained by slow diffusion of methanol vapor into a chloroform solution of **2a**. A suitable crystal was selected, and the X-ray diffraction data were collected on a Bruker D8 Venture diffractometer using a μ-focus Cu Kα radiation source (λ = 1.54178 Å) with collimating mirror monochromators. Data were collected using ω-scans. The structures were solved by direct methods using SHELXT and refined by full-matrix least-squares on F2 with anisotropic displacement parameters for the non-H atoms using SHELXL-2018/3. Structure analysis was aided by use of the programs PLATON[3] and OLEX2.

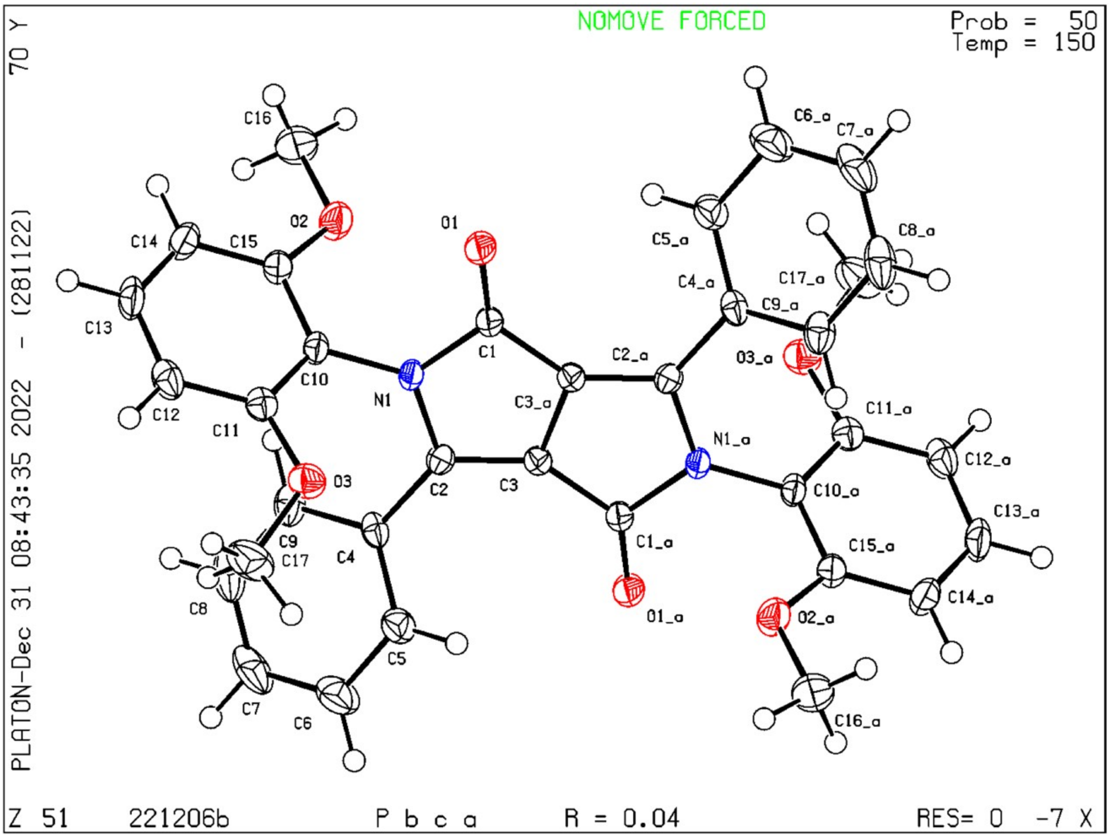

Figure S23. ORTEP drawing of **2a** with the thermal ellipsoids shown at the 50% probability. Nitrogen blue, oxygen red, carbon medium gray, hydrogen white.

Diffraction grade single crystals of **5a** were obtained by slow diffusion of methanol

vapor into an acetonitrile solution of **5a**. A suitable crystal was selected, and the X-ray diffraction data were collected on a Bruker D8 Venture diffractometer using a μ-focus Cu Kα radiation source (λ = 1.54178 Å) with collimating mirror monochromators. Data were collected using ω-scans. The structures were solved by direct methods using SHELXT[1] and refined by full-matrix least-squares on F2 with anisotropic displacement parameters for the non-H atoms using SHELXL-2018/3. Structure analysis was aided by use of the programs PLATON[3] and OLEX2.

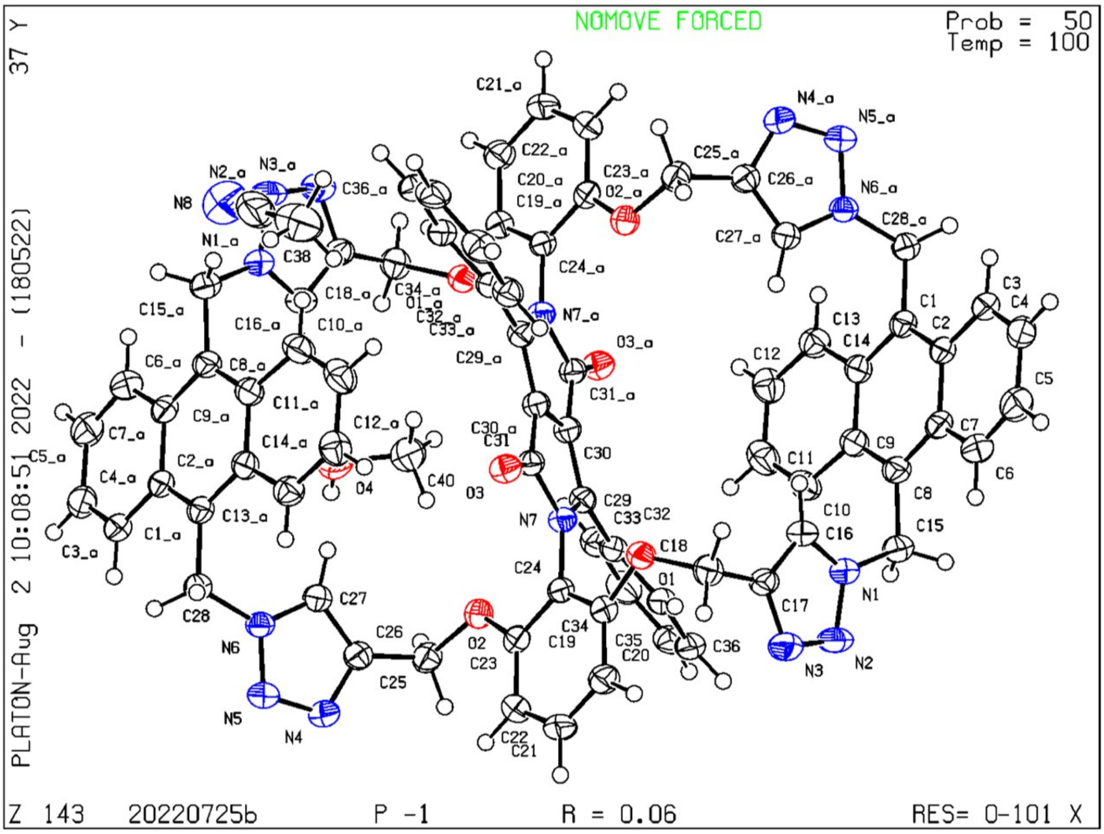

Figure S24. ORTEP drawing of **5a** with the thermal ellipsoids shown at the 50% probability, which contains one methanol and one acetonitrile molecules. Nitrogen blue, oxygen red, carbon medium gray, hydrogen white.

Table S1. Crystal data and structure refinement for **2a** and **5a**.

Identification code	221206b	20220725b
Empirical formula	$C_{34}H_{28}N_2O_6$	$C_{80}H_{66}N_{16}O_8$
Formula weight	560.58	1379.48
Temperature/K	150(2)	100(2)
Crystal system	orthorhombic	triclinic
Space group	P b c a	P -1
a/Å	10.6143(4)	11.1096(7)
b/Å	13.2575(5)	11.2145(6)
c/Å	19.9774(7)	15.2337(9)
α/°	90	68.402(3)
β/°	90	79.501(4)
γ/°	90	69.937(3)
Volume/Å^3	2811.20(18)	1654.20(17)
Z	4	1
ρ_{calc} g/cm^3	1.325	1.385
μ/mm^{-1}	0.746	0.752
F(000)	1176	722
Crystal size/mm^3	0.130 × 0.160 × 0.160	0.020 x 0.050 x 0.080
Radiation	Cu Kα (λ = 1.54178 Å)	Cu Kα (λ = 1.54178 Å)
2Θ range for data collection/°	4.43 to 68.49	3.13 to 66.81
Index ranges	-12<=h<=11, -15<=k<=16, -24<=l<=22	-13<=h<=13, -13<=k<=13, -18<=l<=18
Reflections collected	29349	25142
Independent reflections	2572 [R(int) = 0.0379]	5866 [R(int) = 0.0501]
Data/restraints/parameters	2572 / 0 / 192	5866 / 0 / 472
Goodness-of-fit on F^2	1.181	1.058
Final R indexes [I>=2σ (I)]	R_1 = 0.0377, wR_2 = 0.1001	R1 = 0.0650, wR2 = 0.1861
Final R indexes [all data]	R_1 = 0.0389, wR_2 = 0.1008	R1 = 0.0757, wR2 = 0.1961
Largest diff. peak/hole / e Å^{-3}	0.196 and -0.265	1.178 and -0.687
CCDC number	2233723	2233724

5. Photophysical properties

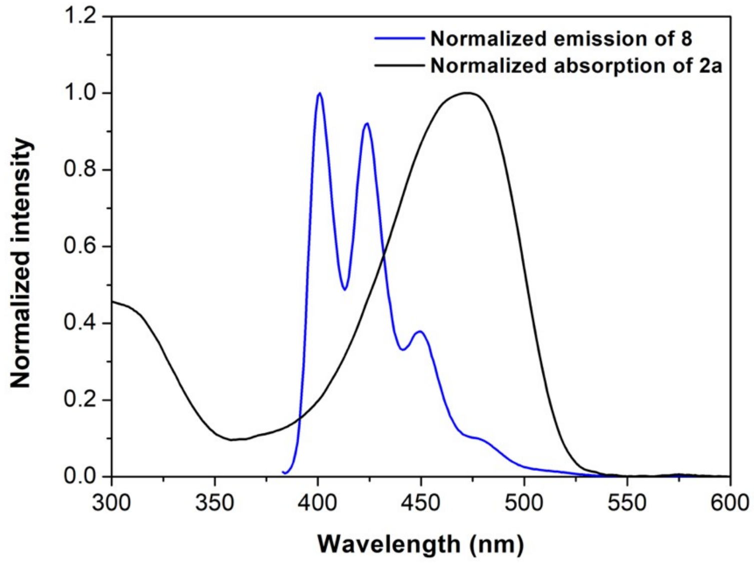

Figure S25. Normalized fluorescence spectrum of compound **8** and UV-Vis absorption spectrum of compound **2a**.

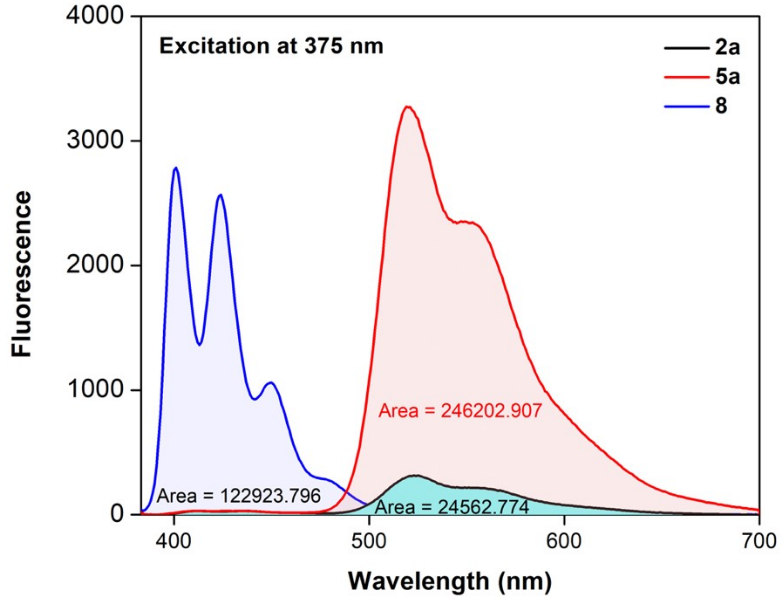

Figure S26. Fluorescence spectra, including relative intensity, of compounds **2a**, **5a**, and **8** in chloroform (10 μM) recorded upon excitation of the anthracene chromophore at 375 nm.

6. Metal ion sensing

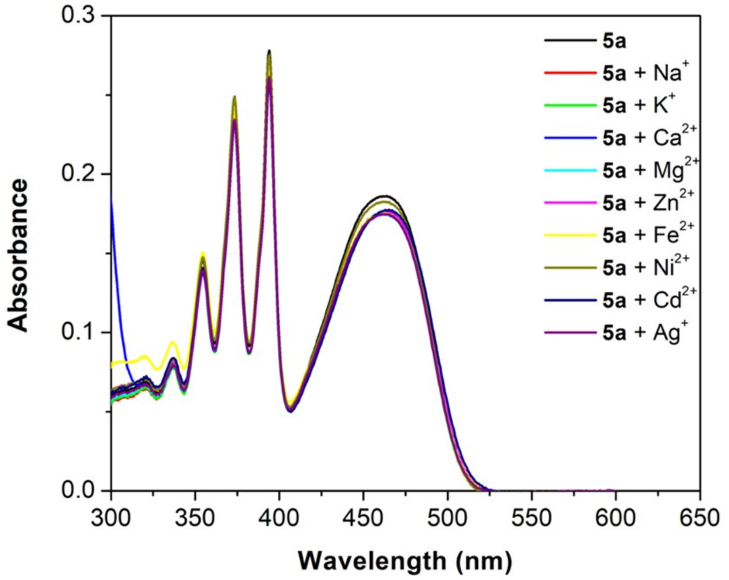

Figure S27. UV-vis absorption spectra of **5a** (10^{-5} M, in CH_3CN) before and after the addition of various metal ion salts, including those of Na^+, K^+, Ca^{2+}, Mg^{2+}, Zn^{2+}, Fe^{2+}, Ni^{2+}, Cd^{2+}, Ag^+ (100 equiv.).

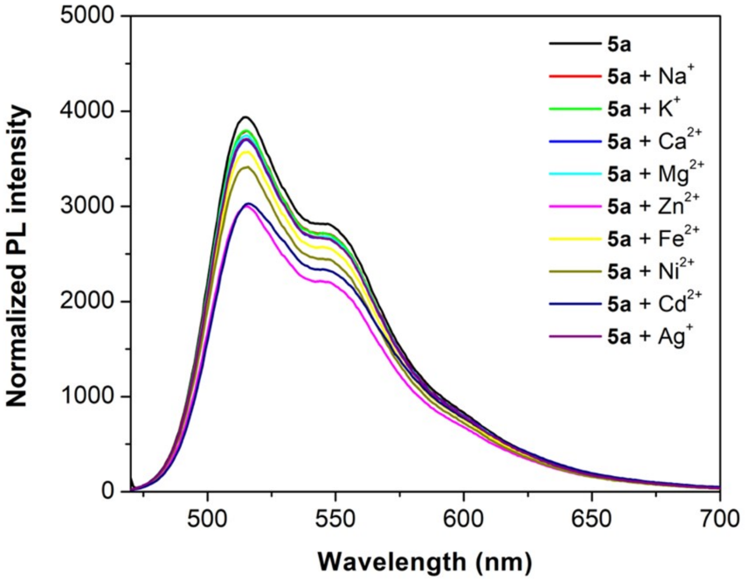

Figure S28. Fluorescence spectra of **5a** (10^{-5} M, in CH_3CN) before and after the addition of various metal ion salts, including those of Na^+, K^+, Ca^{2+}, Mg^{2+}, Zn^{2+}, Fe^{2+}, Ni^{2+}, Cd^{2+}, Ag^+ (100 equiv.).

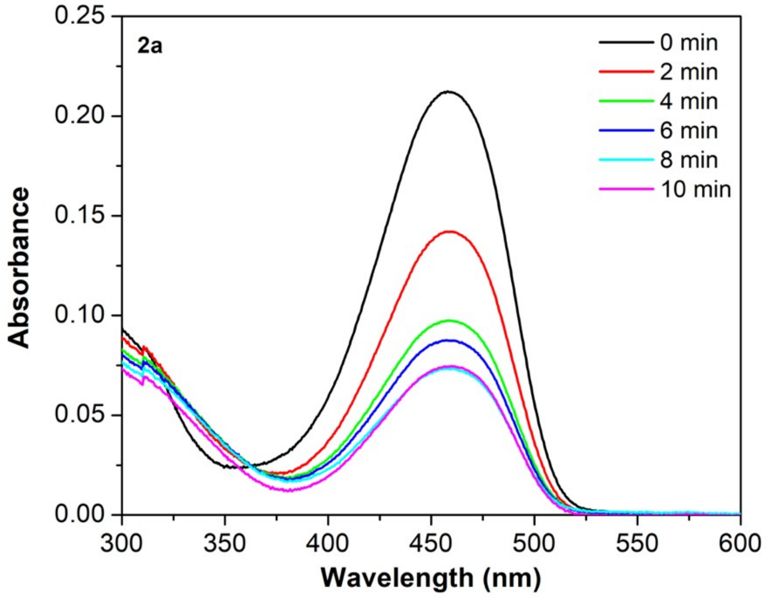

Figure S29. Time dependence evolution of the UV-Vis absorbance spectrum of **2a** (10^{-5} M) after addition of 5 equiv. of a Cu^{2+} salt in acetonitrile.

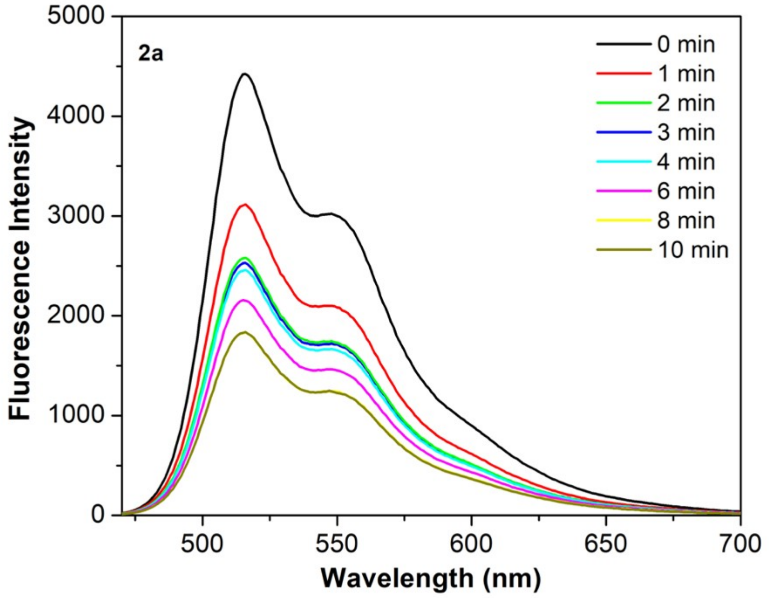

Figure S30. Time dependence of the fluorescence intensity of **2a** (10^{-5} M) recorded after the addition of 5 equiv. of Cu^{2+} in acetonitrile.

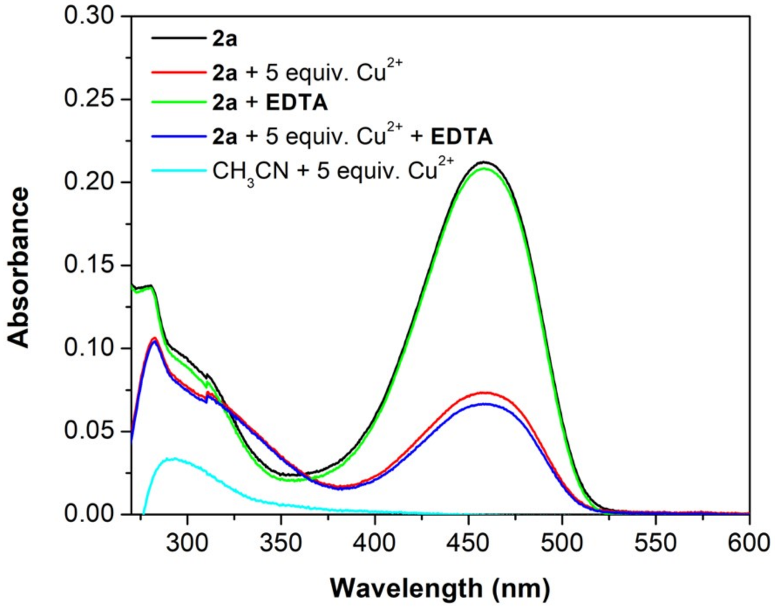

Figure S31. UV-Vis spectra recorded before and after the addition of EDTA to premixed **2a**-Cu^{2+} solutions.

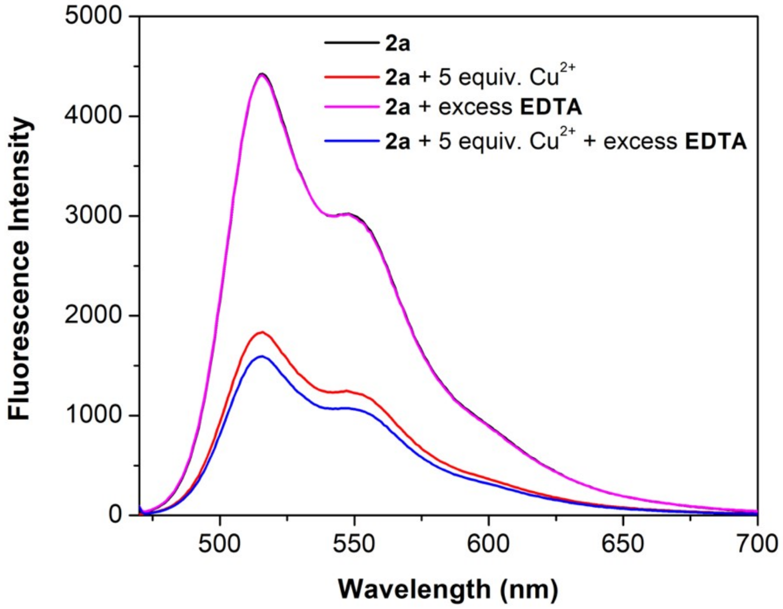

Figure S32. Fluorescence spectra recorded before and after the addition of EDTA to a premixed solution consisting of **2a**-Cu^{2+}.

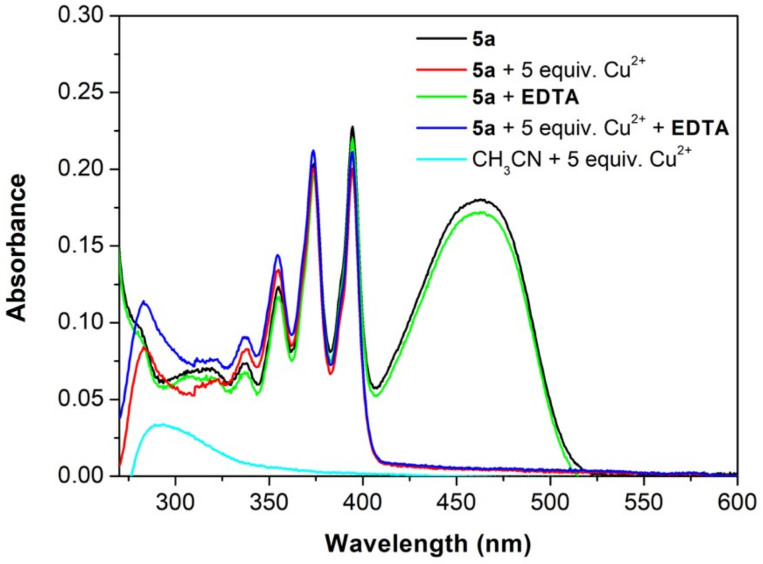

Figure S33. UV-Vis spectra recorded before and after the addition of EDTA to premixed solution of **5a**-Cu^{2+}.

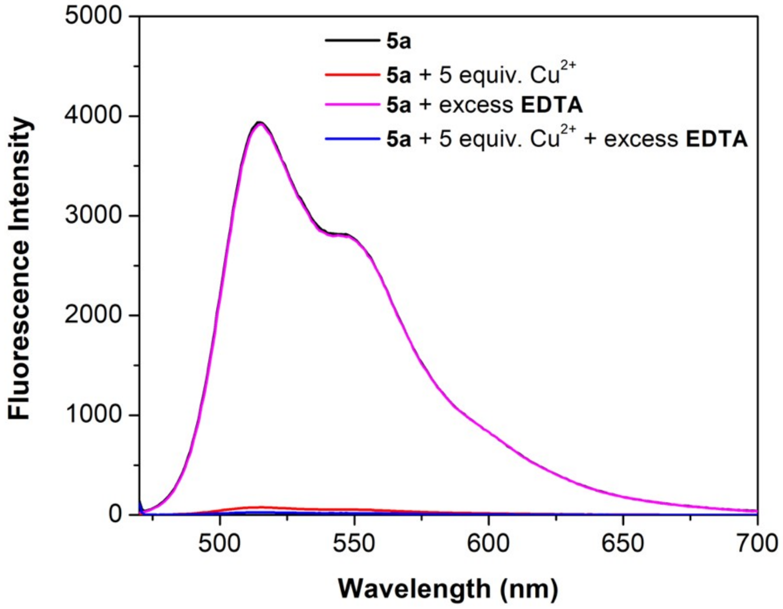

Figure S34. Fluorescence spectra recorded before and after the addition of EDTA to a premixed solution of **5a**-Cu^{2+}.

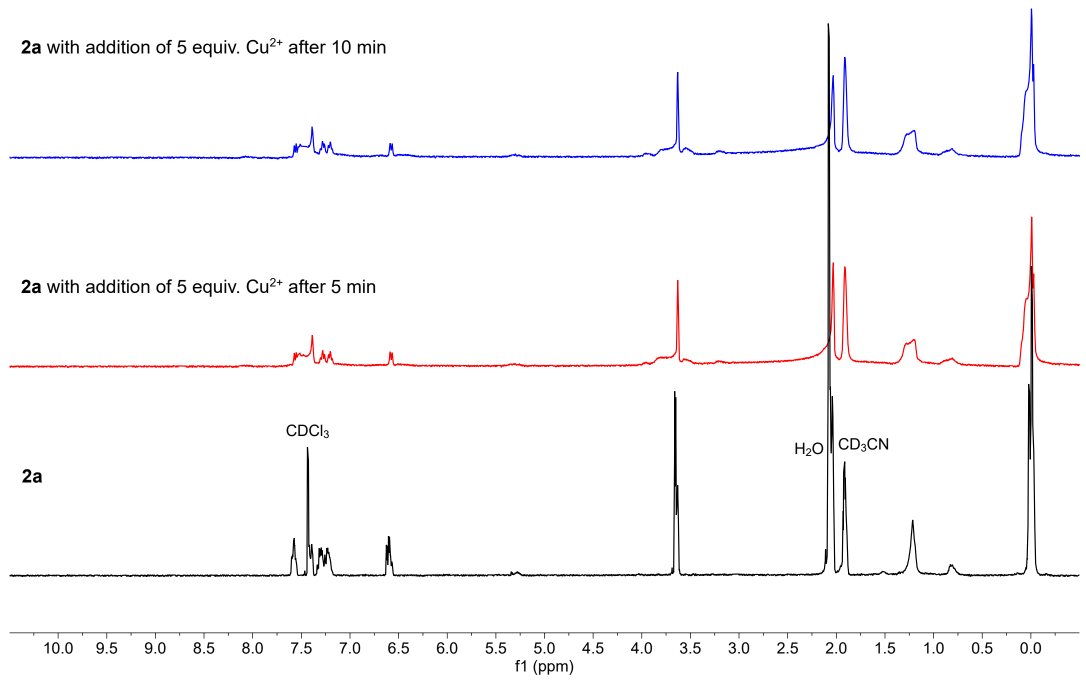

Figure S35. ^{1}H NMR spectra of **2a** recorded before and after the addition of Cu^{2+} in deuterated acetonitrile (containing 25% $CDCl_3$).

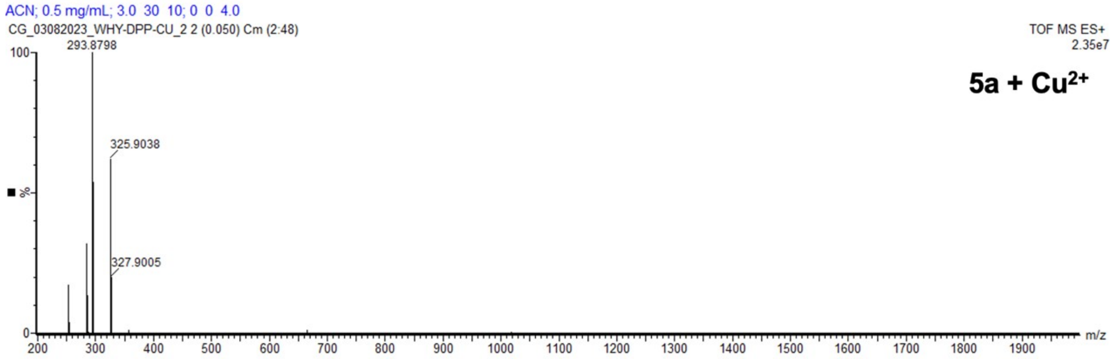

Figure S36. TOF MS spectrum of **5a** after addition of excess Cu^{2+} in acetonitrile. The TOF MS spectrum of **5a** measure in absence of Cu^{2+} shows strong peaks at 1233.23 and 1256.24 corresponding to [**5a** + H]$^{+}$ and [**5a** + Na]$^{+}$, respectively (cf. Fig. S13).

Table S2. Physical properties of the polymers.

Polymer	M_n [a] [kg mol^{-1}]	M_w [a] [kg mol^{-1}]	Dispersity [a]	T_g [^{o}C]
**DPP-Cycle**	25.5	37.1	1.45	336
**DPP-C8C12**	17.7	35.7	2.02	330

[a] Determined by GPC using 1,2,4-trichlorobenzene as the eluent at 160 °C.

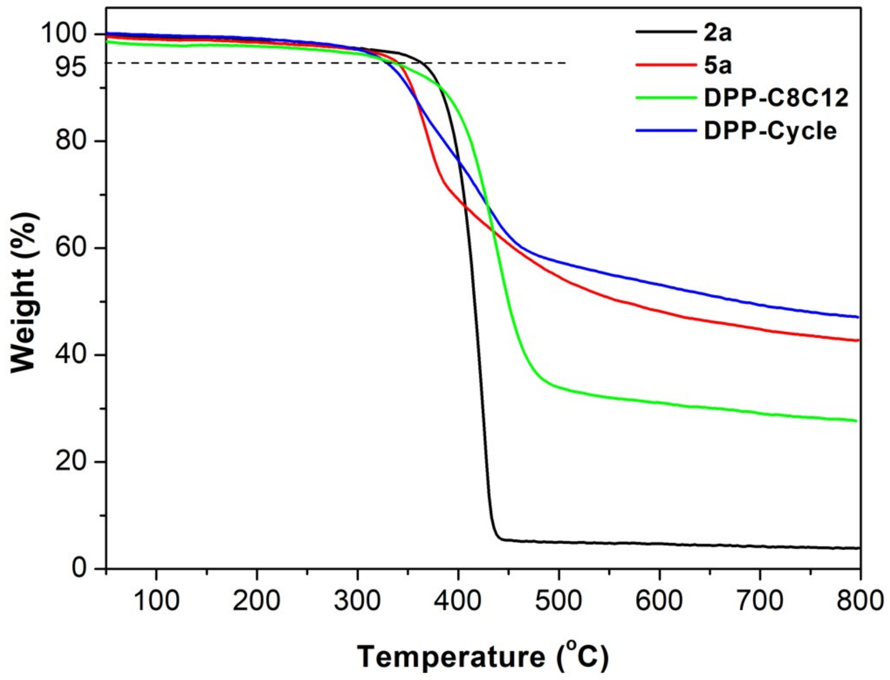

Figure S37. TGA curves recorded under an N_2 atmosphere using a heating rate of 10 °C min^{-1}.

7. Mechanoluminescence

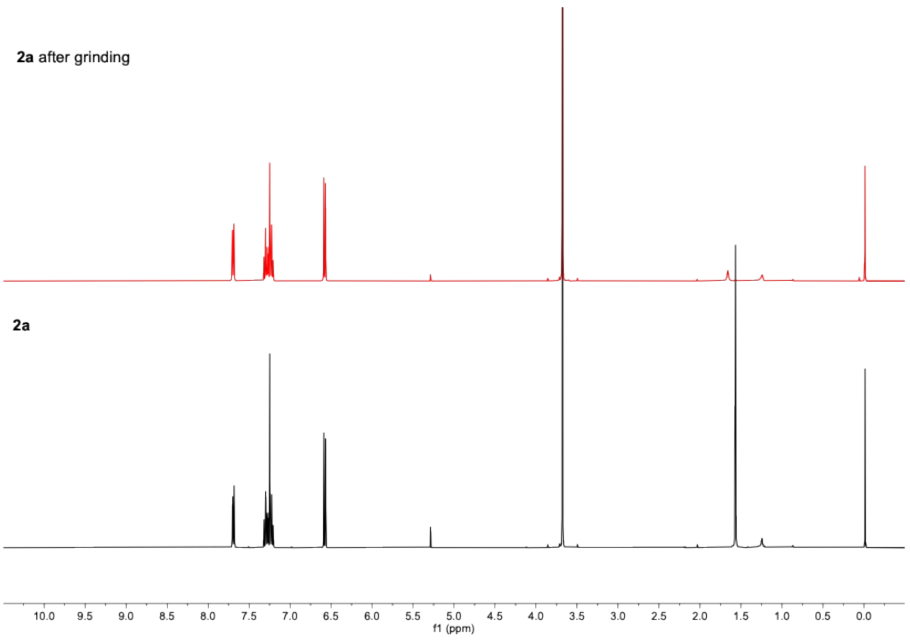

Figure S38. ^{1}H NMR spectra of pristine **2a** (lower trace) and ground **2a** (upper trace) in $CDCl_3$.

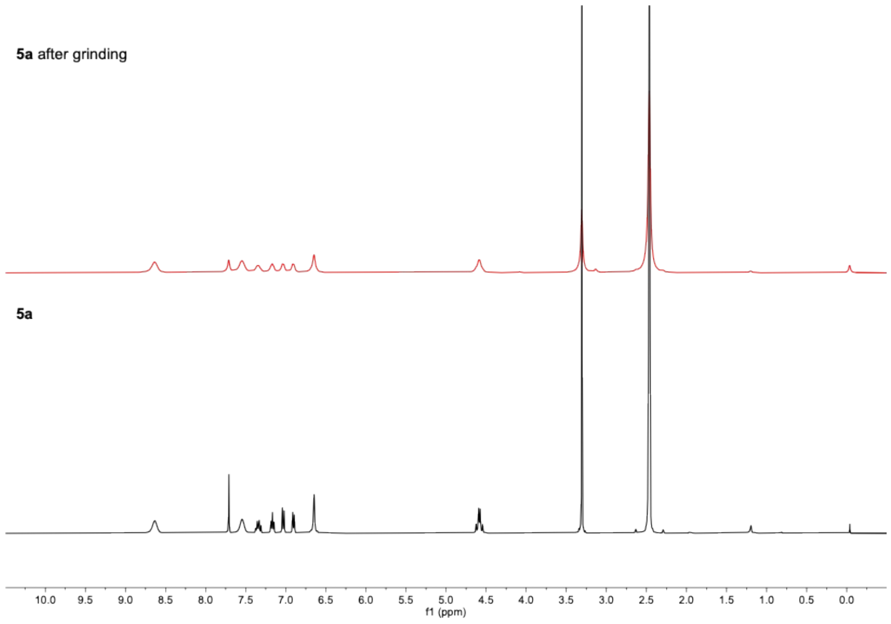

Figure S39. ^{1}H NMR spectra of pristine **5a** (lower trace) and ground **5a** (upper trace) in DMSO-d_6.

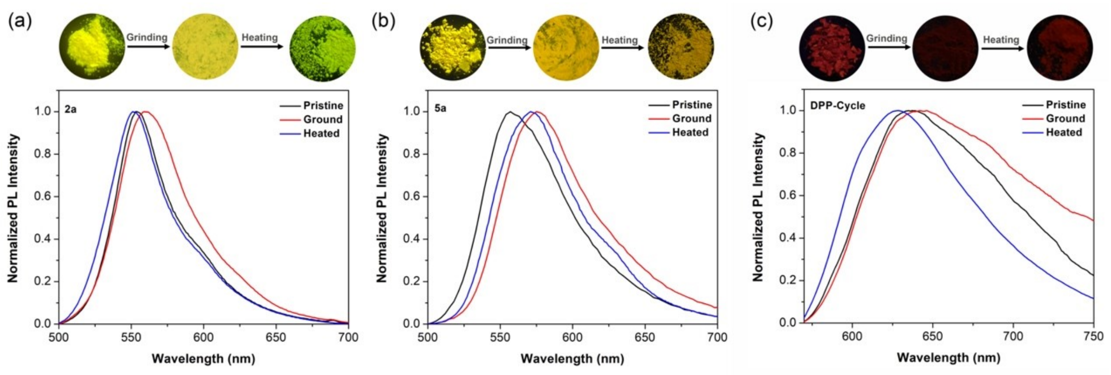

Figure S40. Fluorescence spectra of solid powders of **2a** (a), **5a** (b) and **DPP-Cycle** (c) subject to the indicated treatments. Top: Corresponding fluorescence images taken under a 365 nm ultraviolet lamp.

8. Fluorescence of 5a@silica

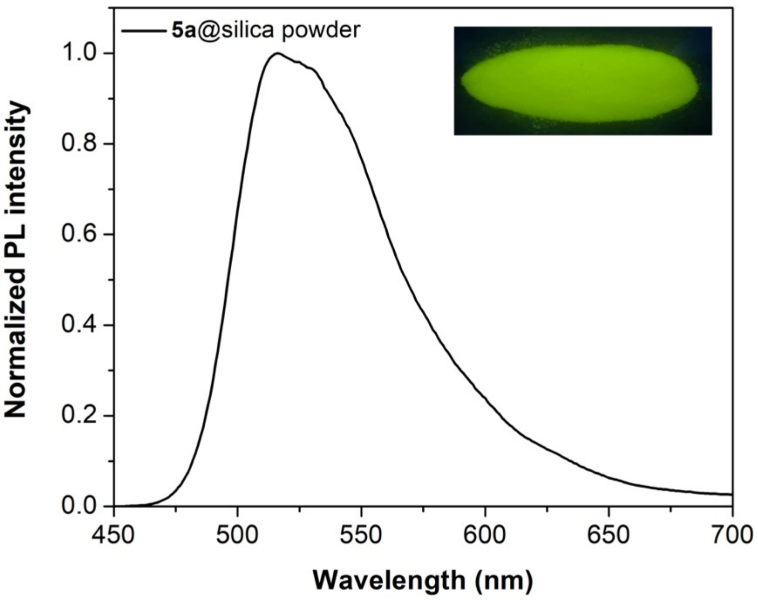

Figure S41. Fluorescence spectrum of **5a**@silica powder. Inset show the fluorescence image of **5a**@silica powder under irradiation at 365 nm using a UV lamp.

9. Supporting References

1. C. Du, S. B. Fu, X, H. Wang, A. C. Sedgwick, Zhen, W.; M. J. Li, X. Q. Li, J. Zhou, Z. Wang, H. Y. Wang and J. L. Sessler, *Chem. Sci*., 2019, **10**, 5699-5704.
2. A. Leventis, J. Royakkers, A. G. Rapidis, N. Goodeal, M. K. Corpinot, J. M. Frost, M. O. Blunt, F. Cacialli and H. Bronstein, *J. Am. Chem. Soc.*, 2018, **140**, 1622-1626.
3. A. L. Kanibolotsky, F. Vilela, J. C. Forgie, S. E. T. Elmasly, P. J. Skabara, K. Zhang, B. Tieke, J. McGurk, C. R. Belton, P. N. Stavrinou and D. D. C. Bradley, *Adv. Mater.*, 2011, **23**, 2093-2097.
